# A new insight on the genus *Pteridium* (Dennstaedtiaceae) in Europe based on a revision in the flora of Slovakia

**DOI:** 10.1186/s40529-024-00423-1

**Published:** 2024-08-14

**Authors:** Mykyta Peregrym, Ingrid Turisová, Peter Turis

**Affiliations:** 1 Ecology and Genetics Research Unit, University of Oulu, P.O. Box 3000, 90014 Oulu, Finland; 2 Department of Landscape Gardening and Ecology, Luhansk Taras Shevchenko National University, Hohol’ Str., 90, Myrhorod, 37600 Poltava Region Ukraine; 3 Department of Biology, Ecology and Environment, Matej Bel University in Banská Bystrica, Tajovského Str., 40, 97401 Banská Bystrica, Slovakia

**Keywords:** Distribution, Fern, Floristic finding, Natural range, Polypodiidae, Pteridoflora

## Abstract

**Background:**

The genus *Pteridium* Gled. ex Scop. was thought to be monotypic with the cosmopolitan species *P. aquilinum* (L.) Kuhn. for many years. However, morphological variations among these plants in different regions have been noted since the 1940’s, leading to the description of new taxa later. Molecular investigations, while not resolving all taxonomical questions within the genus, have highlighted its rich genetic diversity globally, confirming the status of several previously described species and subspecies. This wealth of data has prompted revisions of the *Pteridium* genus in regional floras, with Northern Eurasia serving as a central arena for such studies in the last 30 years. Recent data suggest that the European flora comprises a single species, *P. aquilinum*, with two subspecies, *P. a*. subsp. *aquilinum* and *P. a.* subsp. *pinetorum* (C.N. Page & R.R. Mill) J.A. Thomson. However, their distribution within the continent remains unclear. Therefore, this study aims to elucidate the representation and distribution of *Pteridium* taxa in Slovakia with an attempt to describe natural ranges of *P. aquilinum* subspecies based on herbarium materials and citizen science data within Europe for a whole.

**Results:**

It is confirmed that the genus *Pteridium* is represented by the single species with two mentioned subspecies in the flora of Europe, as well as in the flora of Slovakia. The distribution of these subspecies is mapped in the country. Additionally, we discuss the ranges of these subspecies for a whole, and we evidence the growth of *P.* *aquilinum* subsp. *pinetorum* within the Netherlands, Czechia, Austria, Hungary and Romania for the first time. Also, two new combinations of taxa from the Asian part of Russia are offered.

**Conclusion:**

Our study is a significant contribution in the present knowledge about the distribution and taxonomy of *P. aquilinum*, however it also shows that many questions concerning this taxon and its infraspecific taxa remain open. Additional field investigations and herbarium processing should be carried out for detailed explorations of biological and ecological peculiarities of the mentioned subspecies, and for the clear understanding of their regional distribution. Such explorations also might become a basis for new syntaxonomic revisions.

**Supplementary Information:**

The online version contains supplementary material available at 10.1186/s40529-024-00423-1.

## Background

The Slovak Republic is a relatively small country located in Central Europe, but with a rich flora within the European Union. The study of vegetation cover of the modern territory of Slovakia has been started by Lumnitzer (Lumnitzer 1791), Endlicher (Endlicher 1830) and Reuss (Reuss 1853) since the end of the eighteenth century—the first part of the nineteenth century, and the last sources mention here 4253 taxa of native and alien vascular plants (Medvecká et al. 2012). However, the exploration process of the local flora is actively going on at present: enough many new native and alien species for the country have been recently discovered (Király et al. 2014; Dítě et al. 2019; Kobiv et al. 2022; Eliáš Jr. et al. 2023). Nonetheless, there are a few groups of plants, like leptosporangiate ferns, which are studied sporadically in Slovakia, therefore information about them is rarely updated. So, it was known 44 species of Polypodiidae including their hybrids 57 years ago (Futák et al. 1966), but this number increased until 50 species 32 years later (Marhold and Hindák 1998) that was mostly connected with changes of taxonomic ranks of some subspecies till species. Only one species of ferns, *Asplenium platyneuron* (L.) Britton, Sterns & Poggenb., was discovered in the country from that time (Ekrt and Hrivnák 2010). However, it cannot be said that the exploration of Polypodiidae is over in the Slovak flora, and our new insight on the genus *Pteridium* (Dennstaedtiaceae) is a confirmation of this affirmation.

For a long time, the genus *Pteridium* Gled. ex Scop. was considered in the world flora as monotypic, i.e. with one cosmopolitan species *P. aquilinum* (L.) Kuhn. But, botanists have paid attention to the differentiation of morphological peculiarities of these plants in different regions of our planet since the 40’s of the last century. At that time, Tryon described 2 subspecies and 12 varieties within the species (Tryon 1941). The activation of the study of *Pteridium* started in the end of the 80’s–90’s when several subspecies, varieties and a new species (*P. pinetorum* C.N. Page & R.R. Mill**)** were described in Europe (Page 1989; Page & Mill 1994, 1995). The next stage in *Pteridium* exploration is connected with the results of molecular investigations which have not given answers to all taxonomical questions within the genus yet, but they show its rich genetic diversity around the world that allows separating several species and subspecies from the previous single one (Thomson 2000, 2004; Der et al. 2009; Zhou et al. 2014; Wolf et al. 2019).

All these data have become a basis for revisions of *Pteridium* genus in regional floras, and Northern Eurasia has become a central arena of the processing during last 30 years (Tutin et al. 1993; Tzvelev 2003, 2005, 2010; Thomson 2004; Gureyeva and Page 2008; Frank 2008; Tikhomirov 2009; Tzvelev and Geltman 2012; Zhou et al. 2014; Zając et al. 2019; Wolf et al. 2019; Zenkteler and Nowak 2019; Zenkteler et al. 2022). Moreover, clarification of the species composition of *Pteridium* in the flora of Eastern Europe grew into a heated scientific discussion where from 2 to 4 species of the genus were given at different times (Tzvelev 2003, 2005, 2010; Gureyeva and Page 2008; Gureyeva 2011; Tzvelev and Geltman 2012). Though, there is no final agreed conception yet, according to the last published data of morphological and molecular investigations (Thomson 2000, 2004; Der et al. 2009; Tikhomirov 2009; Zhou et al. 2014; Wolf et al. 2019; Zenkteler and Nowak 2019; Zenkteler et al. 2022) only the single species (*P. aquilinum*) with two subspecies (*P*. *a*. subsp. *aquilinum* (L.) Kuhn and *P. a.* subsp. *pinetorum* (C.N. Page & R.R. Mill) J.A. Thomson) is presented in the flora of Europe. But, the question "how are they distributed within the continent?" remains open.

The described situation made us think about the subspecies composition of *Pteridium* in Central Europe, namely in Slovakia and its some neighboring countries, especially since the two mentioned subspecies of *P.* *aquilinum* were noted for Poland (Zając et al. 2019; Zenkteler and Nowak 2019). As well, *P. aquilinum*, *P. latiusculum* (Desv.) Hieron. and *P. pinetorum* were listed for the flora of the Ukrainian Carpathians (Tzvelev 2005; Vasheka and Bezsmertna 2012). Therefore, we set a goal to figure out which taxa of the genus *Pteridium* are represented in Slovakia, and how it/they is/are distributed within the country. Also, we tried to describe natural ranges of *P. aquilinum* subspecies based on herbarium materials and citizen science data within Europe for a whole.

## Methods

The research was carried out during 2022–2023, but it is based on the results of our long-term field explorations within Slovakia and its neighboring countries, the processing of herbarium materials in *BP*, *BRA*, *SAV*, *SLO*, *ZV* (here and beyond herbarium acronyms are given according to the Index Herbariorum: https://sweetgum.nybg.org/science/ih/), as well as data from, a citizen science platform, *iNaturalist* (https://www.inaturalist.org). The list of checked herbarium specimens and citizen science observations are given in Supplementary materials A.

Nowadays there is no the single consensus on the rank of taxa in the genus *Pteridium*. For example, Eurasian *P. pinetorum* is being considered as a separated species (Page and Mill 1994), or also as a subspecies of *P.* *aquilinum* or *P. latiusculum*: *P. aquilinum* subsp. *latiusculum* (Desv.) Hultér (Karlsson 2000), *P. aquilinum* subsp. *pinetorum* (Thomson 2004; Wolf et al. 2019), *P. aquilinum* subsp. *japonicum* (Nakai) Á.Löve & D.Löve (Zhou et al. 2014) or *P. latiusculum* subsp. *pinetorum* (C.N. Page & R.R. Mill) Fraser-Jenk. (Fraser-Jenkins 1997; Fraser-Jenkins et al. 2015) respectively. No doubt such confusion found its way into the leading taxonomic databases. For example, POWO (https://powo.science.kew.org), and Euro+Med PlantBase (https://europlusmed.org) accept *P. pinetorum* as an independent species, Tropicos (https://www.tropicos.org) gives *P*. *aquilinum* subsp. *japonicum* and/or *P. a.* var. *latiusculum* (Desv.) Underw. ex A. Heller as accepted names for this taxon, but World Flora Online (https://wfoplantlist.org) considers *P. pinetorum, P. aquilinum* subsp. *pinetorum* and *P. a.* var. *pinetorum* (C.N.Page & R.R.Mill) Perestor. as synonyms of *P. latiusculum* subsp. *pinetorum*. Since establishing the status of taxa in the genus *Pteridium* was not the task of our study, we adopted the most reasonable, in our opinion, point of view on this debatable issue based on the results by Wolf et al. (2019) and partially by Der et al. (2009) with Zhou et al. (2014). So, we suggest that only *P. aquilinum* with two subspecies (*P*. *a*. subsp. *aquilinum* and *P. a.* subsp. *pinetorum*) is presented in the flora of Europe. The identification of these *Pteridium* subspecies is based on morphological criteria described in literature (Page and Mill 1994; Frank 2008; Tikhomirov 2009; Zenkteler and Nowak 2019; Zenkteler et al. 2022). However, it is worth to note that we considered these mentioned subspecies on the species level on earlier stages of our research, therefore our *Notae criticae* we left in herbarium collections contain the name “*Pteridium pinetorum* C.N. Page & R.R. Mill” that actually should be accepted as *P. aquilinum* subsp. *pinetorum.*

The maps of currently known locations of *P*. *a*. subsp. *aquilinum* and *P.* *a.* subsp. *pinetorum* in Slovakia was produced by available tools at the website "Simplemappr” (https://www.simplemappr.net). In Supplementary Materials A, Slovak localities of these subspecies of *P. aquilinum* are included into phytogeographic districts according to Futák (Futák 1984), and there are sorted according to the Guidelines for processing the flora of Slovakia (Futák 1973). The classification of sites is based on the actual location of the site and may not always agree with what is stated on the herbarium item. In the case of old historical names of municipalities, we also mention their current names in parentheses.

New taxonomic combinations are offered regarding the rules of the current edition of the International Code of Nomenclature for algae, fungi, and plants (Shenzhen Code), namely Article 41 (Turland et al. 2018).

## Results

The genus *Pteridium* is represented by the single species (*P. aquilinum*) with two subspecies (*P*. *a*. subsp. *aquilinum* and *P. a.* subsp. *pinetorum* in the flora of Slovakia. These subspecies have never been noted for the country either in publications and online databases, therefore to aid in their better identification, we have given the genus system for Slovakia with relevant nomenclature citations, an identification key, and up-to-date information regarding their distribution in the region. Also, some synonyms which have been used in European floras are given additionally to escape misunderstanding.

### Proposed system of *Pteridium* in Slovakia

***Pteridium*** Gled. ex. Scop., Fl. Carniol., ed. 1: 169. 1760, non Raf. 1814 nec (Kütz.) J. Agardh, nom. cons. ≡ *Filix* Ludw., Inst. Regn. Veg., ed. 2: 142. 1757, non Ség. 1754 ≡ *Cincinalis* Gled., Syst. Pl. Stamin. Situ: 290. 1764 ≡ *Pteris* sect. *Ornithopteris* J. Agardh, Recens. Spec. Pter.: 45. 1839 ≡ *Eupteris* Newman in Phytologist 2: 278. 1845 ≡ *Ornithopteris* (J.Agardh) J. Sm., Hist. Fil.: 297. 1875, non Bernh. 1805 ≡ *Filix-foemina* Hill ex Farw. in Amer. Midl. Naturalist 12: 290. 1931

Type: *Pteridium aquilinum* (L.) Kuhn (– *Pteris aquilina* L.).

***Pteridium aquilinum*** (L.) Kuhn in Ascherson & al., Bot. Ost-Afrika (= in Decken, Reisen Ost-Afrika, 3 (3, Bot.)): 11. 1879 ≡ *Pteris aquilina* L., Sp. Pl.: 1075. 1753 ≡ *Cincinalis aquilina* (L.) Gled., Syst. Pl. Stamin. Situ: 290. 1764 ≡ *Eupteris aquilina* (L.) Newman in Phytologist 2: 278. 1845 ≡ *Ornithopteris aquilina* (L.) J.Sm., Hist. Fil.: 298. 1875 ≡ *Filix aquilina* (L.) Woynar in Hedwigia 56: 383. 1915 ≡ *Filix-foemina aquilina* (L.) Farw. in Amer. Midl. Naturalist 12: 290. 1931, nom. superfl.

Lectotype (designated by Tryon 1941): Europe, [illustration] “Filix foemina.” [= “Waldfarn weible.”] in Fuchs, Hist Stirp.: 596 [misprinted “569”]. 1542—Epitype (designated by Thomson 2004): Europe, Hort. Cliff. p. 473, Pteris 6? (*BM* barcode BM000647565 n.v.)

Distribution: throughout Africa, Asia, Europe, and North America, also the range extends to Malesia and Central America. 2n = 104 (Takahashi 1961).

***Pteridium aquilinum*** subsp. ***aquilinum*** in Fl. Europ., ed. 2, 1: 16; Page & Mill, in Bot. J. Scotl. 47, 2: 234. 1995; Page, The Ferns of Britain and Ireland, ed. 2: 349. 1997 ≡ *P.* *aquilinum* (L.) Kuhn var. *typicum*, Tryon, in Rhodora 43, 505: 15. 1941.—Lectotype: the same as for the species.

= *Pteris brevipes* Tausch, in Flora 19 (2): 427. 1836 ≡ *Pteridium aquilinum* (L.) Kuhn subsp. *brevipes* (Tausch.) Wulf, in Fl. Taur. 1: 20. 1927.—Described from Czechia “Herb. Fl. Boh. univ.”; holotype is unknown (Tzvelev 2005).

= *Allosorus tauricus* C. Presl, in Tent. Pterid. 154. 1836, nom. inval. ≡ *Pteridium tauricum* V.I. Krecz. ex Grossh. in Fl. Kavkaza, ed. 2, 1: 35. 1939 ≡ *P.* *aquilinum* (L.) Kuhn subsp. *tauricum* (C.Presl) Gureeva et C.N. Page, in Bot. J. 93, 6: 929. 2008, nom. inval.—Described from the Caucasus; holotype is unknown.

= *Pteridium aquilinum* (L.) Kuhn subsp. *atlanticum* C.N. Page, in Watsonia 17: 431. 1989; Page & Mill, in Bot. J. Scotl. 47, 2: 235. 1995; Page, The Ferns of Britain and Ireland, ed. 2: 349. 1997—Holotype: Scotland: north-east Arran, Clyde Isles (v.c. 100), between the Cock of Arran Laggan, G.R. NR 937511, c. 15 m alt., on Carboniferous limestone, 4 June 1987, C.N. Page 29020, holotype: in *E*, isotypes: in *ABD*, *GL*, *PTH*.

= *P. aquilinum* (L.) Kuhn subsp. *fulvum* C.N. Page, in Page & Mill, Bot. J. Scotl. 47, 1: 139. 1995; Page & Mill, in Bot. J. Scotl. 47, 2: 236. 1995; Page, The Ferns of Britain and Ireland, ed. 2: 356. 1997.—Holotype: Scotland, Perthshire, steep east-facing banks of River Tummel 3 km N of Pitlochry, overlooking Loch Fascally 500 m S of Clunie Power Station, on shallow rocky soil, G.R. NN 915592, 140 m, 17 August 1989, C.N. Page 27053, HM. McHaffie, holotype: in *E*.

Distribution: Western (including Spanish and Portuguese islands in the Atlantic Ocean), Northern (Denmark and southern parts of Norway and Sweden including Gotland), Central, Southern and Eastern (western and central regions of Belarus’, the Crimean Mountains and western regions of Ukraine) Europe, Western Asia (Asia Minor, the Caucasus, and the Alborz).

**Fig. 1:**
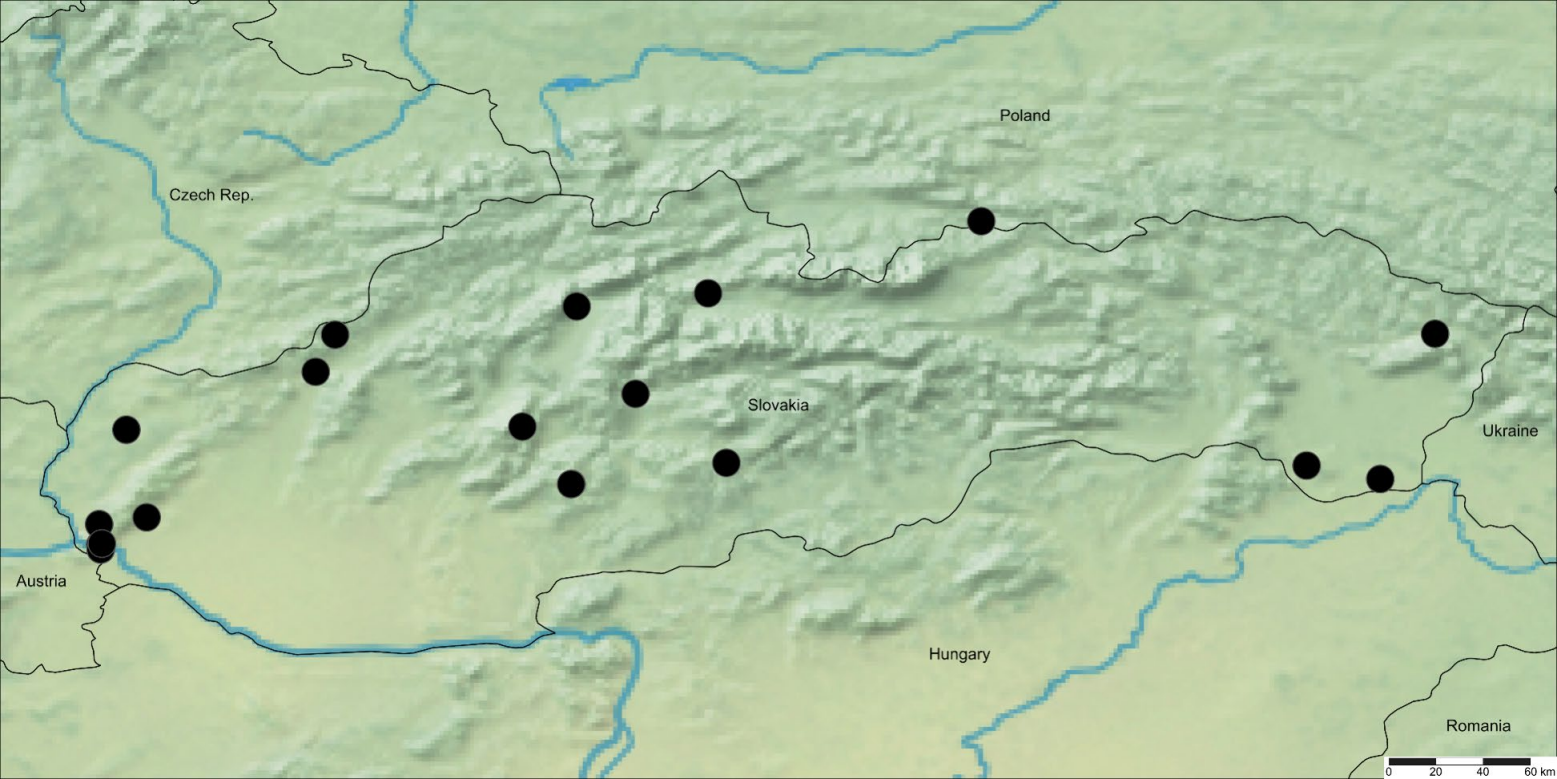
Distribution of *Pteridium aquilinum* (L.) Kuhn subsp. *aquilinum* in Slovakia (“●”—confirmed localities)

Distribution in Slovakia: mainly southern parts of Slovakia in Pannonian and Praecarpatian floristic regions, occasionally also further north in Eucarpatian floristic regions (more details in Supplementary Materials A, as well as Fig. 1.).

***Pteridium aquilinum*** subsp. ***pinetorum*** (C.N. Page & R.R. Mill) J.A. Thomson in Telopea 10: 798. 2004 ≡ *P. pinetorum* C.N. Page & R.R. Mill in Bot. J. Scotland 47: 140. 1995; Page & Mill, in Bot. J. Scotl. 47, 2: 239. 1995; Page in The Ferns of Britain and Ireland, ed. 2: 363. 1997 ≡ *P. latiusculum* subsp. *pinetorum* (C.N. Page & R.R. Mill) Fraser-Jenk. in New Sp. Syndr. Indian Pteridol.: 220. 1997 ≡ *P. aquilinum* (L.) Kuhn var. *pinetorum* (C.N. Page & Mill) Perest., in N.I. Shorina and O.N. Perestoronina, Proc. Intl Bracken Group Conference, Manchester 1999, Bracken Fern: Toxicity, Biology and Control Ch. 7:51. 1999 (publ. August 2000)—Holotype: Scotland: E Inverness-shire, Rothiemurchus Forest near Loch an Eilein, G.R. NH 896092, 4 June 1983, C.N. Page 17049 (holotype in *E* barcode E00026882 n.v.; isotypes in *ABD*, *GL*, *PTH*; cited by Page (1989: p. 431) as voucher of *P. aquilinum* subsp. *latiusculum*, with locality differently spelled "Loch an Eilean").

= *Pteridium aquilinum* (L.) Kuhn var. *osmundaceum* Christ in Beitrage Krypt. Schweiz 1, 2: 54. 1900 ≡ *P. pinetorum* C.N. Page & R.R. Mill subsp. *osmundaceum* (Christ) C.N. Page, in Bot. J. Scotl. 47: 140. 1995. – Syntypes: from eastern Switzerland (Graubunden: Alvaneu) and N Italy (several localities near Bórmio, Lombardia) (Page & Mill 1994).

Notes: some previous authors named this taxon as *P. aquilinum* (L.) Kuhn subsp. *latiusculum* (Desv.) C.N. Page (Page 1989); *P. aquilinum* subsp. *latiusculum* (Desv.) Hultér (Karlsson 2000), *P. latiusculum* (Desv.) Hieron. ex Fries (Tzvelev 2005; Tzvelev and Geltman 2012), but these names might be correctly applied to a subspecies of *P. aquilinum* from North America described from Newfoundland (Canada), not occurring in Slovakia and Europe in general. As well, the name, *P. aquilinum* (L.) Kuhn subsp. *japonicum* (Nakai) Á. Löve & D. Löve, was used by Zhou & al. (Zhou et al. 2014) for European specimens of this taxon, but this subspecies is distributed only in the North-Eastern Asian region with its lectotype from Japan. Thus, the best name for European plants noted under the mentioned taxa is *Pteridium aquilinum* subsp. *pinetorum*.

Distribution: Western, Northern, Central and Eastern Europe (exclude the Crimean Peninsula), Asian part of Russia, Kazakhstan, Mongolia, and China.

**Fig. 2:**
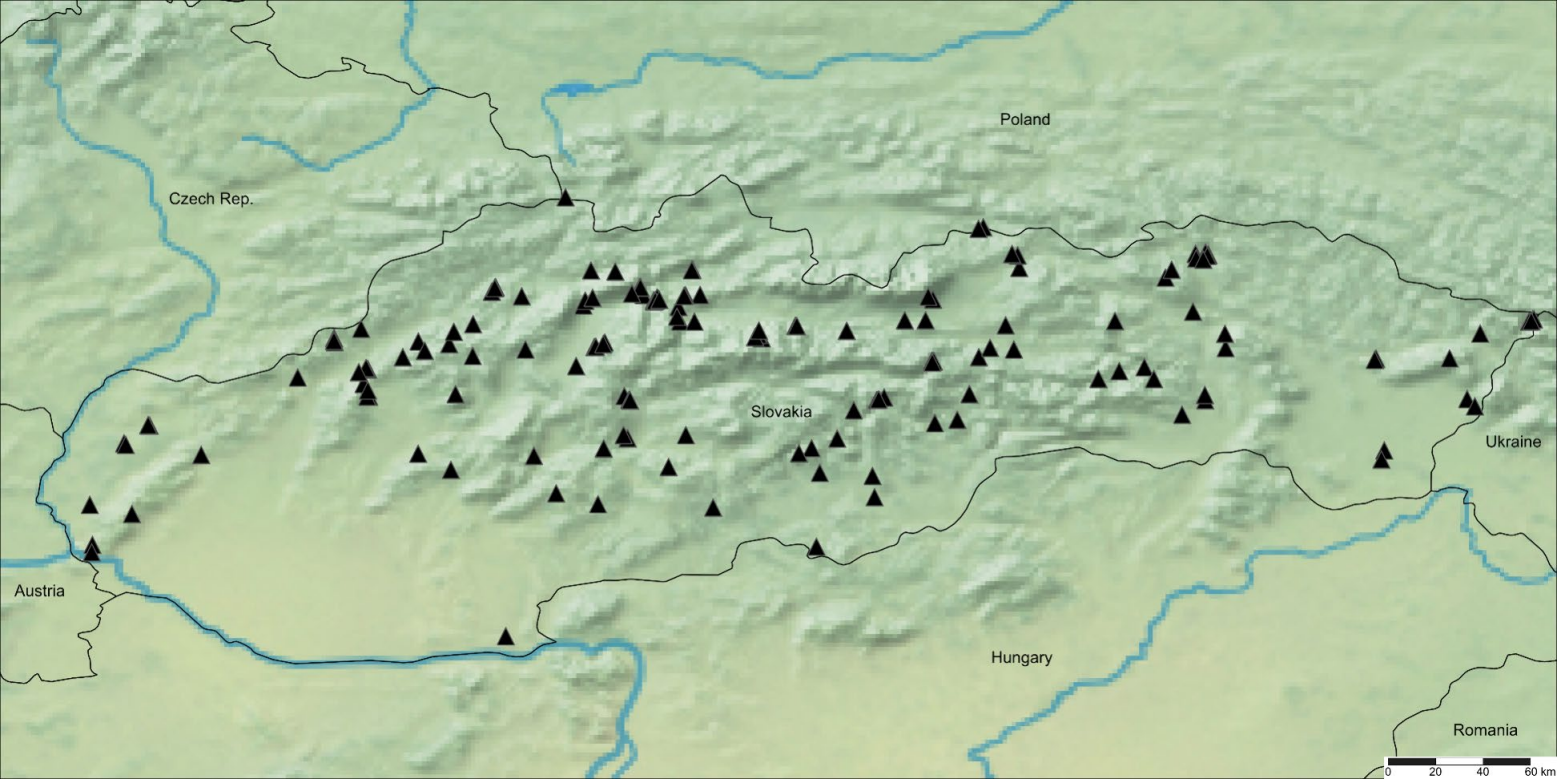
Distribution of *Pteridium aquilinum* (L.) Kuhn subsp. *pinetorum* (C.N. Page & R.R. Mill) J.A. Thomson in Slovakia (“▲”—confirmed localities)

Distribution in Slovakia: whole territory, except of agriculture used Pannonian parts of country (more details in Supplementary Materials A, as well as Fig. 2.).

### The identification key for *Pteridium* subspecies for Slovakia and neighbouring countries

**Fig. 3:**
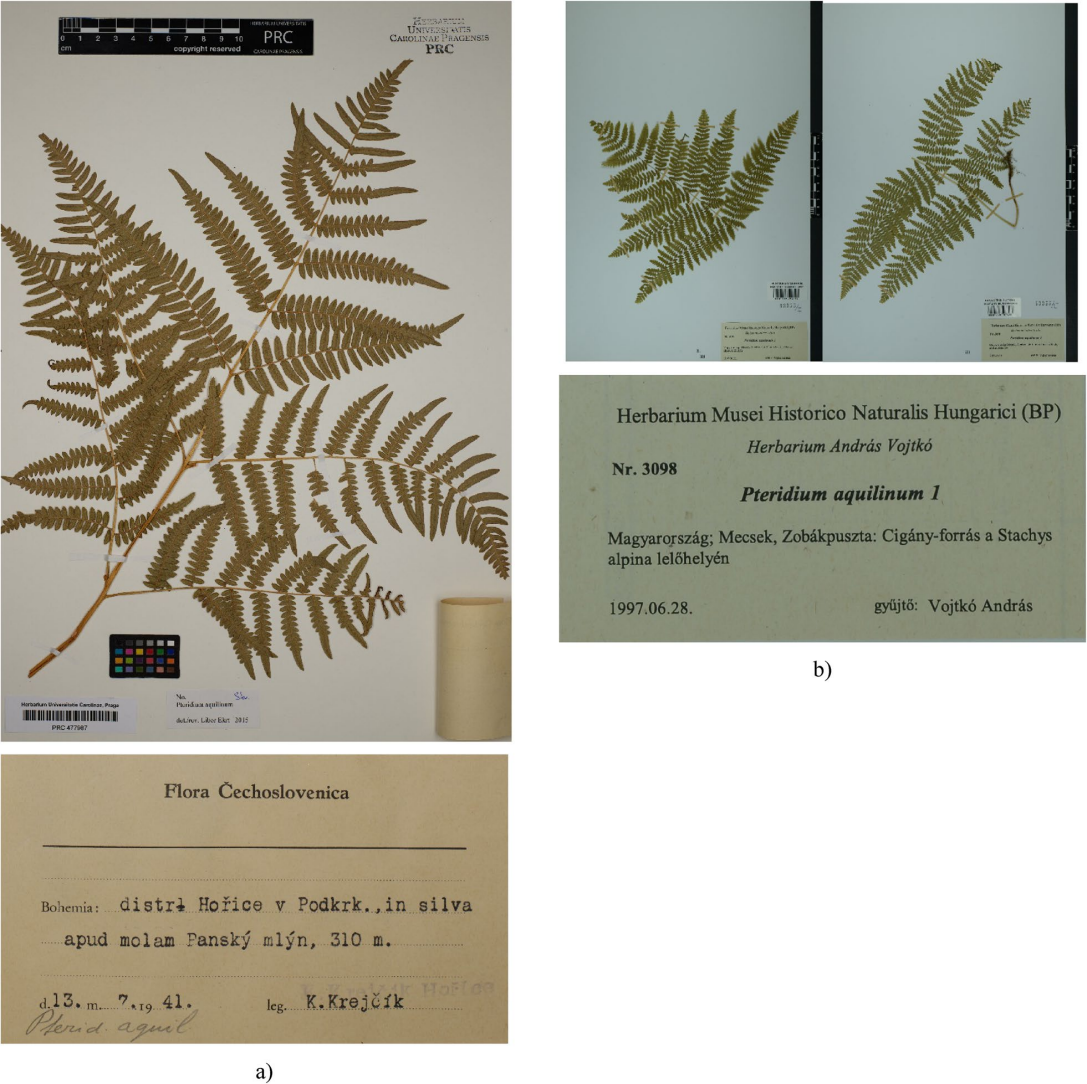
*P. aquilinum* subsp. *aquilinum*: **a** a specimen from Czechia (*PRC* #477987, Charles University, Prague, Czechia); **b** the single individual of divided into two herbarium sheets collected in Hungary (*BP* #53355/I and #53355/II, Hungarian Natural History Museum, Budapest, Hungary)

**Fig. 4:**
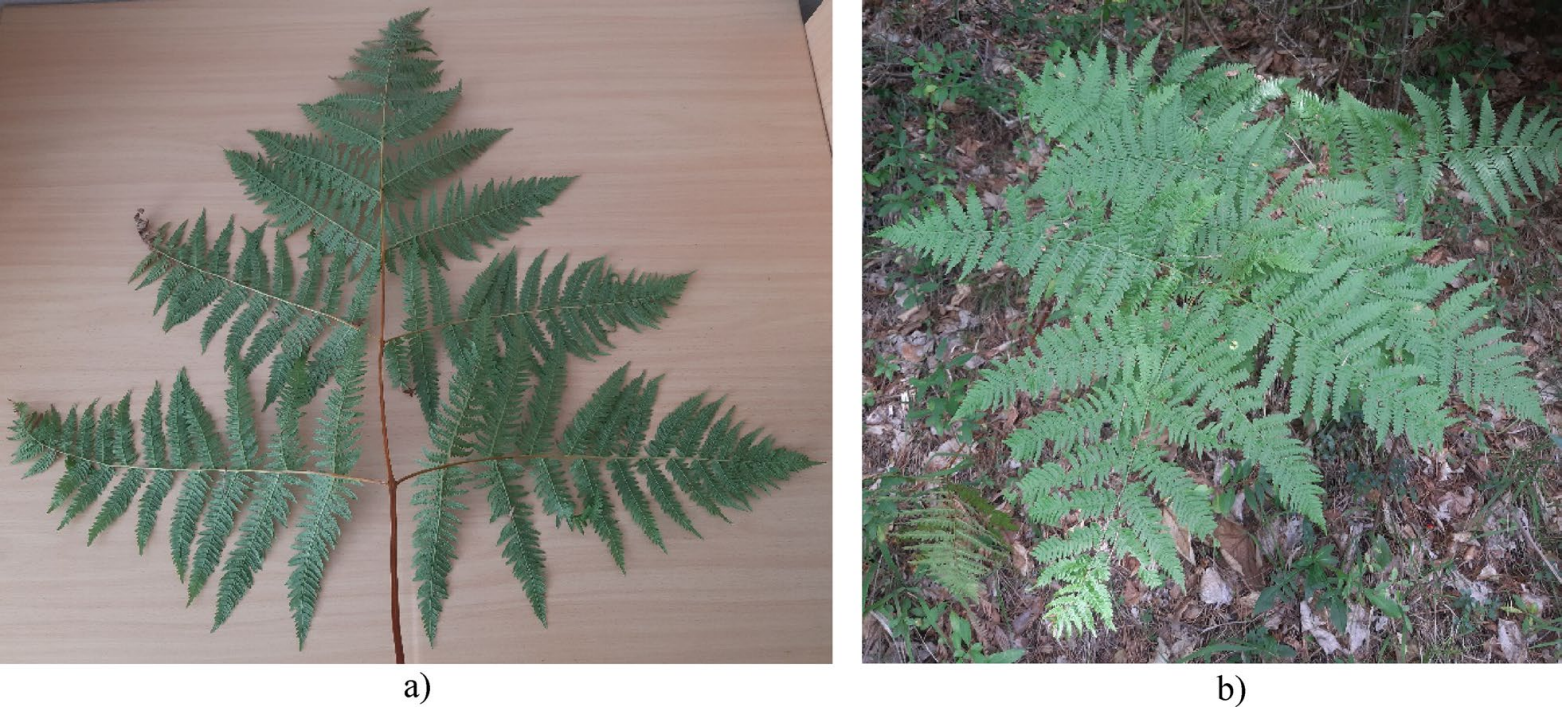
*P. aquilinum* subsp. *pinetorum*: **a** a general view of the fern frond from the underside; **b** in a natural habitat near Banska Bystrica (Slovakia)

1. Leathery frond lamina; rachis upright; the second pair of pinnae are the longest; pinnules elongate, sessile, acute at apex; pinnae dissection 2–3 (rare 3–4); right angle joined pinna to rachis ………… ***P.*** ***aquilinum subsp. aquilinum*** (Fig. 3).

* Straw frond lamina; rachis arcuate; the first pair of pinnae are the longest; pinnules ovate on stipites, rounded at apex; pinnae dissection 2; acute angle between rachis and pinnae …………… ***P.*** ***aquilinum subsp. pinetorum*** (Fig. 4).

## Discussion

**The subspecies of** ***Pteridium aquilinum*** **in Slovakia.** The occurrence of two subspecies of *P. aquilinum* in the flora of Slovakia is expected, because *P. a.* subsp. *pinetorum* or *P. pinetorum* has been previously reported in neighboring regions of Poland (Zając et al. 2019; Zenkteler and Nowak 2019) and Ukraine (Tzvelev 2005; Tzvelev and Geltman 2012; Vasheka and Bezsmertna 2012) for several times earlier. However, an unpredicted moment is that *P.* *aquilinum* subsp. *pinetorum* is a common subspecies for the country based on the processed herbarium and citizen science data, meanwhile *P. a.* subsp. *aquilinum* is significantly rarer here. Likely, the latter subspecies should be regarded as a rare taxon in Slovakia. However, this suggestion requires additional field investigations. As well, detailed studies of the distribution of the *P.* *aquilinum* subspecies should be implemented in the country in the near future, ideally with the use of herbarium data from Austria and Czechia. Moreover, such investigations are needed to establish the common ranges of *P. a.* subsp. *aquilinum* and *P. a.* subsp. *pinetorum* in details, because presently there is a lack of relevant information.

Also, biology and ecology of these subspecies are not studied well enough in Europe yet. It means that a lot of data exists concerning of *P. aquilinum* aggr. (Marrs and Watt 2006), however this information only partly describes biological and ecological peculiarities of its subspecies (Gureyeva and Page 2008). According to our field visual observations in Slovakia, habitats of *P. a.* subsp. *pinetorum* are coniferous or mixed forests mostly on sandy soils with the domination or co-domination of the Scots pine (*Pinus sylvestris* L.) and their natural ecotones, but this subspecies can sometimes grow in an anthropogenic transformed places as forest slashes or roadsides. While our current focus is on planning and next conducting specialized studies on the ecological-coenotic peculiarities of *P. a.* subsp. *pinetorum,* our existing data align with information available from various regions of Europe (Page 1989, p. 19; Frank 2008; Vasheka and Bezsmertna 2012). At the same time, *P.* *a.* subsp. *aquilinum* mostly prefers open and wetter habitats: diverse deciduous forests (with the domination or co-domination of *Fagus sylvatica* L., *Betula pendula* Roth., *Quercus* L. species etc.), meadows and their ecotones. However, cases of the joint growth of both *Pteridium aquilinum* subsp. *aquilinum* and *P. a.* subsp. *pinetorum* within the boundaries of the single biotope are already known (Bridges et al. 1998), although they have not yet been registered in Slovakia. This phenomenon is also observed for other closely related taxa of the *Pteridium* genus (Dosdall and David 2023). Nevertheless, the biology and ecology of the studied subspecies require additional careful research to detail them.

**Images of the subspecies of * Pteridium aquilinum* in identification guides and floras**. An intriguing aspect arises when examining how *Pteridium aquilinum* is depicted and labeled in scientific literature in Slovakia and some European countries. For instance, the “Flora of Slovakia” and the “Flora of the Czech Republic” present images of a typical *P. a.* subsp. *pinetorum*, but they are labeled simply as *P. aquilinum* (Futák et al. 1966; Hejný and Slavík 1997). A similar situation is observed in the “Ecoflora of Ukraine” (Didukh 2000). A photo of *P. a.* subsp. *aquilinum* is featured in an article about *P. aquilinum* in the “Red Data Book of Moldova” (Duca 2015). At the same time, a typical *P. a.* subsp. *aquilinum* is drawn at a Hungarian plant identification guide, but it is labeled simply as *P. aquilinum* (Király et al. 2011). It is also depicted and labeled in the Flora (Săvulescu 1952), as well as in the latest plant identification guide of Romania (Sârbu et al. 2013). A notable example is found in a periodical edition of the Excursion flora of Germany. A typical *P. a.* subsp. *pinetorum* was published there, but it was identified as *P. aquilinum* (Rothmaler et al. 1995). However, in a more recent edition (Jäger et al. 2017), separate pictures of both subspecies with correct identifications have been included. As well, there are some cases when only a part of a frond or a leaf of plants from the genus *Pteridium* is imagined in publications (Raciborski and Szafer 1919; Josifović 1970; Hess et al. 1976; Demiri 1983; Dostál and Červenka 1991). Such pictures or photos might be informative in the context of comparing with other genera of ferns, but they do not allow seeing significant differences among infraspecific taxa of *P. aquilinum* clearly.

Thus, this underscores the need for meticulous attention when preparing images of *P. aquilinum* and its subspecies for future editions of regional floras, lists of rare and protected plants, field guides, and similar publications, especially taking into account the information about ranges of the subspecies of *P. aquilinum* given below.

**Fig. 5:**
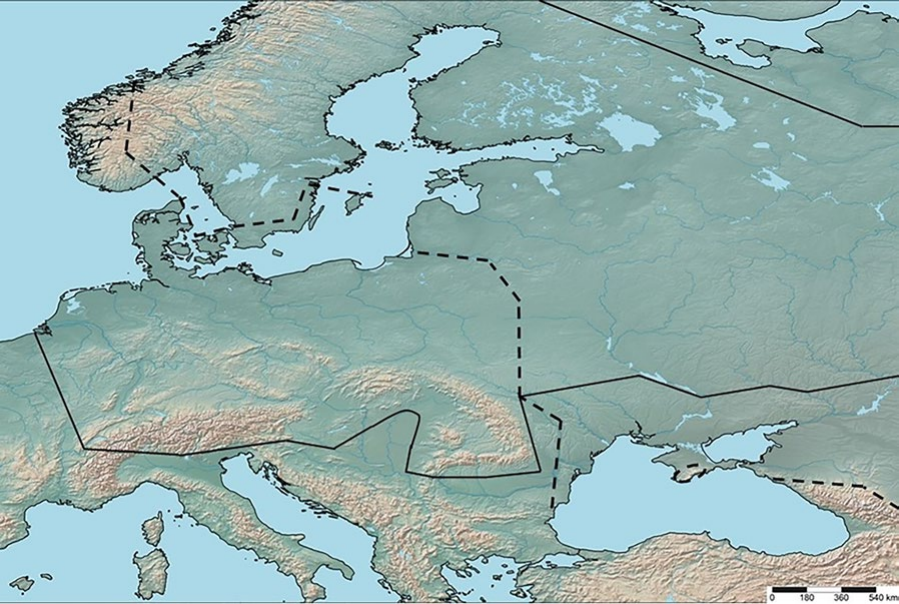
The borders of distribution of *Pteridium aquilinum* subsp. *aquilinum* (“---”) and *P. a.* subsp. *pinetorum* (“**–**”) in Europe

**Ranges of the subspecies of** ***Pteridium aquilinum.*** Taking into account the latest published data (Karlsson 2000; Thomson 2000; Tzvelev 2005, 2010; Thomson et al. 2005; Gureyeva and Page 2008; Der et al. 2009; Parfenov 2009; Gureyeva 2011; Tzvelev and Geltman 2012; Zhou et al. 2014; Kurtto et al. 2019; Wolf et al. 2019) and our obtained results, it was established that *P. a.* subsp. *aquilinum* should now be classified as a European-West Asian subspecies, which was previously erroneously noted for Africa (Thomson et al. 2005). However, it is presumed to be absent in most of Fennoscandia and the Baltic countries, except for the southern parts of Norway and Sweden, as well as possibly Lithuania. The eastern boundary of the subspecies' distribution extends from the central regions of Belarus (Parfenov 2009; Tikhomirov 2009), through the western regions of Ukraine, including the Carpathians (Tzvelev 2005, 2010; Tzvelev and Geltman 2012; Vasheka and Bezsmertna 2012), continuing through Moldova (Duca 2015), Romania and Bulgaria, avoiding steppe and forest-steppe areas (Fig. 5). It then extends to the north and northwest regions of Western Asia, covering Asia Minor, the Caucasus, and the Alborz. There is also an isolated fragment of its range within the Crimean Mountains (Tzvelev 2005, 2010; Tzvelev and Geltman 2012; Vasheka and Bezsmertna 2012).

The most part of *P. a.* subsp. *pinetorum* range is located in Forest and Steppe-Forest zones of Asian part of Russia, going a little into the eastern regions of Kazakhstan and the northern regions of Mongolia, until contact zones with *P. a.* subsp. *japonicum* (Nakai) Á.Löve & D.Löve and *P. a.* subsp. *wightianum* (Wall. ex J.Agardh) W.C.Shieh at Far East and in China (Tzvelev 2005, 2010; Gureyeva and Page 2008; Gureyeva 2011; Zhou et al. 2014; Wolf et al. 2019). The distribution of *P. a.* subsp. *pinetorum* is studied only fragmentary in Europe at present. The presence of this subspecies is confirmed on the territory of the British Isles, Scandinavian and Baltic countries, Germany, Switzerland, northern Italy, Poland, Belarus, Ukraine, as well as Forest and Forest-Steppe zones of European Russia, including some isolated localities at the northern slopes of the Great Caucasus (Page and Mill 1994; Karlsson 2000; Thomson 2004; Tzvelev 2005, 2010; Gureyeva and Page 2008; Frank 2008; Tikhomirov 2009; Gureyeva 2011; Tzvelev and Geltman 2012; Vasheka and Bezsmertna 2012; Kurtto et al. 2019; Wolf et al. 2019; Zenkteler and Nowak 2019). Also, it presumably is presented in the flora of Austria and Liechtenstein (Frank 2008). In any way, the western and southern borders of *P. a.* subsp. *pinetorum* distribution stays still unclear. Results of our research allow drawing an indicative line describing the boundary of the range of the subspecies in Europe, although without any doubt it should be clarified after regional studies (Fig. 5). So, this border runs approximately between the Steppe and Forest-Steppe zones in Ukraine, then including the Carpathians crossing the Pannonian Basin to the Alps, and after that probably through northern Croatia and Slovenia (where this taxon has never previously been noted), northern Italy and Switzerland to Luxembourg and the Netherlands. Nevertheless, we have no confirmed data on the occurrence of *P. a.* subsp. *pinetorum* in France, Luxembourg and Belgium, except for information from several taxonomic databases as POWO (https://powo.science.kew.org/taxon/urn:lsid:ipni.org:names:17565260-1) and Euro+Med Plantbase (https://www.europlusmed.org/cdm_dataportal/taxon/46d929da-2995-4b6b-a8ba-250b1b0a216c), so perhaps its western border might be run a little east to Germany. But, it has been found an evidence of the presence of *P. a.* subsp. *pinetorum* in the Netherlands: Nijmegen, písek v borových lesících (sand in pine forests), 31.V.1935, Leg. Ptý, *SAV*, Rev.: *Pteridium pinetorum* C.N. Page & R.R. Mill, 12.10.2022, M. Peregrym. Also, herbarium samples from Austria, Czechia, Hungary and Romania which clearly confirm the growth of *P. a.* subsp. *pinetorum* in these countries were discovered during our study (Supplementary materials B).

**Notes regarding taxon naming:** ***P. aquilinum*** **subsp.** ***pinetorum***

It is important to explain why we use the name *P. aquilinum* subsp. *pinetorum* instead of *P. aquilinum* subsp. *latiusculum*. At least, this question appeared several times from your colleagues during the preparation of our manuscript. Therefore, this moment needs clear clarifications. Actually, we support A. Thomson’s opinion (Thomson 2004) which is presently confirmed by latest molecular data (Zhou et al. 2014; Wolf et al. 2019). Namely, *P. aquilinum* subsp. *latiusculum* (Desv.) Hultér, described from Canada as *Pteris latiuscula* in 1827, is exclusively distributed within North America (Canada, USA, Mexico), therefore the using of this name for the European flora, in particular by Karlsson for the Flora Nordica (Karlsson 2000), was mistaken. Thus, this name is incorrectly to apply to European subspecies of *Pteridium aquilinum*.

However, there is one more name, *P.* *aquilinum* (L.) Kuhn subsp. *latiusculum* (Desv.) C.N. Page, which could be a priority one for the discussed taxon. But, it appeared as a new combination with a new, lower, rank from *Pteris latiuscula* (Page 1989), because the combination *Pteridium aquilinum* subsp. *latiusculum* (Desv.) Hultén was not accepted initially, because Hultén made no reference to the basionym (Thomson 2004). However, Hultén’s combination became to consider valid later under the International Code of Botanical Nomenclature (Saint Louis Code) Arts 33.2 and 33.3 (as it was published before 1 January 1953) (Thomson 2004). Moreover, Page and Mill described *P. pinetorum* as a new species for science a little bit later, and *P. aquilinum* (L.) Kuhn subsp. *latiusculum* (Desv.) C.N. Page has already been specified as a synonym in its description (Page & Mill 1994). Therefore, Thomson had to offer a new combination for this taxon on the subspecies level in his revision of the genus *Pteridium* (Thomson 2004) to clearly distinguish subspecies of *P. aquilinum* in North America and Europe. That is why we consider *P. aquilinum* subsp. *pinetorum* as a most suitable name in the context of our research.

**Infraspecific structure of** ***P. aquilinum*** **subsp**. ***pinetorum***. As noted above, the presently accepted subspecies, *P. aquilinum* subsp. *pinetorum*, has been considered in diverse taxonomic statuses and under different names in Eurasia earlier: as the mentioned one (Thomson 2004; Wolf et al. 2019), as *P. pinetorum* (Page and Mill 1994; Gureyeva and Page 2008; Tzvelev 2010; Tzvelev and Geltman 2012), as *P. latiusculum* (Tzvelev 2005), as *P*. *aquilinum* subsp. *latiusculum* (Page 1989; Karlsson 2000), as *P*. *aquilinum* subsp. *japonicum* (Zhou et al. 2014), and as others less significant in the context of the issue under discussion. Moreover, three subspecies of *P. pinetorum* have been described: *P. p.* subsp. *pinetorum*, *P. p.* subsp. *sibiricum* Gureeva et C.N. Page and *P. p.* subsp. *sajanense* Stepanov (Gureyeva and Page 2005, 2008; Stepanov 2012). The first one occurs in Europe, the second one—in Siberia, and the last one locally meets in the Western Sayan Mountains (Gureyeva and Page 2008; Stepanov 2012). However, the relevancy of the description of *P. p.* subsp. *sibiricum* as a new subspecies caused controversy (Tzvelev 2010; Tzvelev and Geltman 2012). Nevertheless, taking into account the latest molecular data which have not confirmed the species’ status of *P. pinetorum* (Thomson 2000, 2004; Der et al. 2009; Zhou et al. 2014; Wolf et al. 2019)**,** and it is accepted as a subspecies of *P. aquilinum* today, consequently it is not correct to consider the mentioned infraspecific taxa on the level of subspecies of an unaccepted species. Therefore, we offer new taxonomic combinations for them as varieties:

*Pteridium aquilinum* subsp. *pinetorum (C.N. Page & R.R. Mill) J.A. Thomson* var. *sibiricum (Gureeva et C.N. Page) M. Peregrym,* *comb. et stat. nov.*

Basionym: *Pteridium pinetorum* C.N. Page & R.R. Mill subsp. *sibiricum* Gureeva et C.N. Page, in Sist. zam. po mater. Herb. im. P.N. Krylova [Syst. notes on materials from the P.N. Krylov Herb. of the Tomsk State Univ.], 95: 22. 2005.

Type citation: «Oкpecтнocти г. Hoвocибиpcкa, cocнoвый лec c opлякoвым пoкpoвoм, 19 VIII 2004. И.И. Гypeeвa»

Type: Russian Federation, surroundings of the city of Novosibirsk (*TK*)

***Pteridium aquilinum*** **subsp**. ***pinetorum (C.N. Page & R.R. Mill) J.A. Thomson*** **var.** ***sajanense (Stepanov) M. Peregrym,*** *comb. et stat. nov.*

Basionym: *Pteridium pinetorum* C.N. Page & R.R. Mill subsp. *sajanense* Stepanov, in Sist. zam. po mater. Herb. im. P.N. Krylova [Syst. notes on materials from the P.N. Krylov Herb. of the Tomsk State Univ.], 105: 12. 2012

Type citation: «Кpacнoяpcкий кpaй, Epмaкoвcкий p-н (Зaпaдный Caян), ypoчищe Ocинoвcкиe кocoгopы, зaпaдный cклoн, близ Ocинoвcкoгo бoлoтa, бepeзняк opлякoвый. 23.07.2010. H.B. Cтeпaнoв»

Type: Russian Federation, Krasnoyarskiy krai, Ermakovsky district (the Western Sayan), tract Osinovskie kosogory, western slope, near Osinovskoe swamp (holotype—*KRSU*; isotypes—*TK*, *NS*, *LE*).

Considering the high morphological variability in *Pteridium* populations in the Western Sayan Mountains, as well as the presence of mixed populations of the mentioned intraspecific taxa without clear boundaries of their distribution (Tzvelev 2010; Stepanov 2012), such a taxonomic decision seems absolutely justified.

## Conclusions

Thus, it is established that the genus *Pteridium* is represented by the single species with two subspecies in the flora of Europe, as well as in the flora of Slovakia: *P. aquilinum* subsp. *aquilinum* and *P. a.* subsp. *pinetorum*. Also, ranges of these subspecies were clarified, mentioning *P. a.* subsp. *pinetorum* for the Netherlands, Czechia, Slovakia, Austria, Hungary and Romania for the first time. Besides, new varieties are offered for two taxa from the Asian part of Russia. At the same time, our study shows that many questions according *P. aquilinum* taxa in Europe and Asia remain open. Additional field investigations and processing of herbarium collections should be carried out for detailed explorations of biological and ecological peculiarities of the mentioned subspecies, as well as for the clear understanding of their regional distribution. Such explorations also might become a basis for new syntaxonomic revisions, because *P. aquilinum* is a diagnostic species for many vegetation communities. Finally, our study underlines the importance of herbarium collections at the time when researchers cannot use a lot of literature data without checking the original specimens of plants.

### Supplementary Information

Supplementary Material 1. Known locations of *Pteridium aquilinum* (L.) Kuhn within Slovakia: a) *P. aquilinum* subsp. *aquilinum*, b) *P. aquilinum* subsp. *pinetorum* (C.N. Page & R.R. Mill) J.A. Thomson

Supplementary Material 2. Confirmed locations of *Pteridium aquilinum* subsp. *pinetorum* (C.N. Page & R.R. Mill) J.A. Thomson in Austria, Czechia, Hungary and Romania

## Data Availability

Data will be made available on request.

## References

[CR1] Bridges KM, Ashcroft CJ, Sheffield E (1998) Population analysis of the type localities of some recently recognised taxa of British *Pteridium* (Dennstaedtiaceae: Pteridophyta). The Fern Gazette 15:205–213

[CR2] Demiri M (1983) Flora ekskursioniste e Shqipërisë. Shtëpia Botuese e Librit Shkollor, Tiranë

[CR3] Der JP, Thomson JA, Stratford JK, Wolf PG (2009) Global Chloroplast Phylogeny and Biogeography of Bracken (*Pteridium*; Dennstaedtiaceae). Amer J Bot 96:1041–1049 21628254 10.3732/ajb.0800333

[CR4] Didukh YP (ed) (2000) Ekoflora Ukraïni (= Ecoflora of Ukraine). Vol. 1. Lycopodiophyta, Equisetophyta, Polypodiophyta, Pinophyta. Fitosociocentr, Kyiv

[CR5] Dítě Z, Dítě D, Feráková V (2019) *Eleusine indica* (L.) Gaertn., new species of the adventive flora of Slovakia. Thaiszia J Bot 29(1):77–84

[CR6] Dosdall JD, David AS (2023) Coexistence of Two Species of Bracken (*Pteridium*) in a Narrow Zone of Range Overlap. Amer Fern J 113:237–248. 10.1640/0002-8444-113.4.237 10.1640/0002-8444-113.4.237

[CR7] Dostál J, Červenka M (1991) Vel’ký kl’úč na určovanie vyšších rastlín. 1. Slovenské Pedagogické Nakladatel’stvo, Bratislava

[CR8] Duca G (ed) (2015) Cartea roșie a Republicii Moldova: = Red book of the Republic of Moldova. Știința, Chișinău, Republica Moldova

[CR9] Ekrt L, Hrivnák R (2010) *Asplenium platyneuron*, a new pteridophyte for Europe. Preslia 82:357–364

[CR10] Eliáš P Jr, Májeková J, Hegedüšová K et al (2023) New alien vascular plants of Slovakia: records from 2008–2021. BioInvasions Rec 12:1–30. 10.3391/bir.2023.12.1.01 10.3391/bir.2023.12.1.01

[CR11] Endlicher S (1830) Flora Posoniensis. Joseph Lander, Posonii

[CR12] Euro+Med PlantBase. https://europlusmed.org/. Accessed 24 January 2024

[CR13] Frank D (2008) Man sieht nur, was man kennt. Nicht beachtete indigene Taxa der Gattungen *Pteridium* und *Urtica*. Mitt Florist Kart Sachsen-Anhalt 13:29–40. 10.21248/mfk.177 10.21248/mfk.177

[CR14] Fraser-Jenkins CR (1997) New species syndrome in Indian pteridology and the ferns of Nepal. International Book Distributors, Dehra Dun, India

[CR15] Fraser-Jenkins CR, Kandel DR, Pariyar S (2015) Ferns and fern-allies of Nepal. National Herbarium and Plant Laboratories, Godawari, Nepal

[CR16] Futák J (1973) Smernice pre spracúvanie Flóry Slovenska [Guidelines for Processing the Flora of Slovakia]. In: Špániková A (ed) Bot. Práce. Botanický ústav SAV. Botanický ústav SAV, Bratislava, pp 131–166

[CR17] Futák J (1984) Fytogeografické členenie Slovenska [Phytogeographical differentiation of Slovakia]. In: Bertová L (ed) Flóra Slovenska IV/1. Veda, Bratislava, pp 418–419

[CR18] Futák J, Jasičová M, Schidlay E (1966) Flóra Slovenska. Vydavatel’stvo Slovenskej akadémie vied, Bratislava.

[CR19] Gureyeva II (2011) To the taxonomy of the genus *Pteridium* Gled. ex Scop. in Russia. In: Materials of the International scientific conference dedicated to the 110^th^ anniversary of AA Uranov. Kostroma State University, Kostroma, pp 114–118

[CR20] Gureyeva II, Page CN (2005) On the question of the systematic position of the bracken in Siberia. Syst Notes Mater Krylov Herbarium Tomsk State Univ 95:18–26

[CR21] Gureyeva II, Page CN (2008) The genus *Pteridium* (Hypolepidaceae) in the Northen Eurasia. Bot J 93:915–934

[CR22] Hejný S, Slavík B (eds) (1997) Kvetena Ceské Republiky. 1. Academia, Praha

[CR23] Index Herbariorum. https://sweetgum.nybg.org/science/ih/. Accessed 21 January 2024

[CR24] Hess HE, Landolt E, Hirzel R (1976) Flora der Schweiz und angrenzender Gebiete. 1: Pteridophyta bis Caryophyllaceae, 2., durchges. Aufl. Birkhäuser, Basel Stuttgart

[CR25] iNaturalist. https://www.inaturalist.org. Accessed 21 January 2024

[CR26] Jäger EJ, Müller F, Ritz C et al (eds) (2017) Rothmaler—Exkursionsflora von Deutschland, Gefäßpflanzen: Atlasband. Springer Berlin Heidelberg, Berlin, Heidelberg

[CR27] Josifović M (ed) (1970) Flora SR Srbije. Srpska akademija nauka i umetnosti, Beograd

[CR28] Karlsson T (2000) *Pteridium* Gled. ex Scop. In: Karlsson T (ed) Flora Nordica. 1: Lycopodiaceae to Polygonaceae, Royal Swedish Academy of Sciences. Bergius Foundation, Stockholm, pp 43–47

[CR29] Király G, Virók V, Molnár VA (eds) (2011) Új magyar füvészkönyv: Magyarország hajtásos növényei: ábrák. Aggteleki Nemzeti Park Igazgatóság, Jósvafő

[CR30] Király G, Eliáš P, Dítě D (2014) Two thermophilic alien species new to the flora of Slovakia. Thaiszia J Bot 24:125–134

[CR31] Kobiv Y, Koutecký P, Štech M, Pachschwöll C (2022) First records of *Calamagrostis purpurea* (Poaceae) in the Carpathians, a relict species new to the flora of Slovakia, Ukraine, and Romania. Biologia 77:2459–2468. 10.1007/s11756-022-01083-x 10.1007/s11756-022-01083-x

[CR32] Kurtto A, Lampinen R, Piirainen M, Uotila P (2019) Checklist of the vascular plants of Finland: = Suomen putkilokasvien luettelo. LUOMUS, Helsinki

[CR33] Lumnitzer S (1791) Flora Posoniensis. Siegfried Lebrecht, Lipsiae

[CR34] Marhold K, Hindák F (eds) (1998) Zoznam nižšich a vyššich rastlin Slovenska: = Checklist of non-vascular and vascular plants of Slovakia. VEDA, Bratislava

[CR35] Marrs RH, Watt AS (2006) Biological Flora of the British Isles: *Pteridium aquilinum* (L.) Kuhn. J Ecol 94:1272–1321. 10.1111/j.1365-2745.2006.01177.x 10.1111/j.1365-2745.2006.01177.x

[CR36] Medvecká J, Kliment J, Májeková J et al (2012) Inventory of the alien flora of Slovakia. Preslia 84:257–309

[CR37] Page CN (1989) Three subspecies of bracken, *Pteridium aquilinum* (L.) Kuhn, in Britain. Watsonia 17:429–434

[CR38] Page CN, Mill RR (1994) Scottish bracken (*Pteridium*): new taxa and a new combination. Bot J Scotland 47:139–140. 10.1080/03746609408684824 10.1080/03746609408684824

[CR39] Page CN, Mill RR (1995) The taxa of Scottish bracken in a European perspective. Bot J Scotland 47:229–247. 10.1080/03746609508684831 10.1080/03746609508684831

[CR40] Parfenov VI (ed) (2009) Flora Belarusi : sosudistye rastenija (= Flora of Belarus’. Vascular Plants). Vol. 1. Lycopodiophyta. Equisetophyta. Polypodiophyta. Ginkgophyta. Pinophyta. Gnetophyta. Belaruskaja navuka, Minsk

[CR41] POWO: Plants of the World Online. https://powo.science.kew.org. Accessed 24 January 2024

[CR42] Raciborski M, Szafer W (eds) (1919) Flora Polska—Rośliny naczyniowe Polski i ziem ościennych. Tom I: Paprotniki, iglaste i jednoliścienne. Polska Akademja Umiejętności, Kraków

[CR43] Reuss G (1853) Května Slovenska [Flora of Slovakia]. Tiskem Františka Lorbera, Banská Štiavnica

[CR44] Rothmaler W, Jäger E, Werner K (1995) Exkursionsflora von Deutschland: Atlasband, 9., durchgeseh. und verb. Aufl. Gustav Fischer, Jena Stuttgart

[CR45] Sârbu I, Ştefan N, Oprea A (2013) Plante vasculare din Romania: determinator ilustrat de teren. Ed. Victor B. Victor, Bucureşti

[CR46] Săvulescu T (ed) (1952) Flora Republicii Populare Române. Editura Academiei Republicii Populare Române, București

[CR47] Simplemappr. https://www.simplemappr.net. Accessed 21 January 2024

[CR48] Stepanov NV (2012) New subspecies of *Pteridium pinetorum* C.N. Page et R.R. Mill (Hypolepidaceae) from the Western Sayan Mountains. Syst Notes Mater Krylov Herbarium Tomsk State Univ 105:8–14

[CR49] Takahashi C (1961) Chromosome study on induced apospory in the bracken fern. La Kromosomo 48:1602–1605

[CR50] Thomson JA (2000) Morphological and genomic diversity in the genus *Pteridium* (Dennstaedtiaceae). Ann Bot 85:77–99. 10.1006/anbo.1999.1101 10.1006/anbo.1999.1101

[CR51] Thomson JA (2004) Towards a taxonomic revision of *Pteridium* (Dennstaedtiaceae). Telopea 10:793–803

[CR52] Thomson JA, Chikuni AC, Mcmaster CS (2005) The taxonomic status and relationships of bracken ferns (*Pteridium*: Dennstaedtiaceae) from sub-Saharan Africa. Bot J Linn Soc 148:311–321. 10.1111/j.1095-8339.2005.00413.x 10.1111/j.1095-8339.2005.00413.x

[CR53] Tikhomirov VN (2009) Morphological variability of *Pteridium* (Hypolepidaceae) in Belarus. Bot J 94:1159–1171

[CR54] Tropicos. https://www.tropicos.org. Accessed 24 January 2024

[CR55] Tryon RM (1941) A revision of the genus *Pteridium*. Rhodora 43(1–31):37–67

[CR56] Turland N, Wiersema J, Barrie F, et al (eds) (2018) International Code of Nomenclature for algae, fungi, and plants. Koeltz Botanical Books

[CR57] Tutin TG, Burges NA, Chater AO et al (eds) (1993) Flora Europaea: Vol.1 Psilotaceae to Platanaceae, 2nd edn. Cambridge University Press, Cambridge

[CR58] Tzvelev NN (2003) De Genere *Dryopteris* Adans. (Dryopteridaceae) in Europa Orientali. Nov Syst Plan Vasc 35:7–20

[CR59] Tzvelev NN (2005) The genus *Pteridium* (Hypolepidaceae) in the Eastern Europe and the Northern Asia. Bot J 90:891–896

[CR60] Tzvelev NN (2010) About species of the genus *Pteridium* Gled. ex Scop. (Hypolepidaceae) in Russia. Bull Mosk Obsh Isp Prir Otd Biol 115:73–76

[CR61] Tzvelev NN, Geltman DV (eds) (2012) Conspectus Florae Europae Orientalis. Tovarishchestvo nauchnykh izdaniĭ KMK, Sankt-Petersburg/Moskow

[CR62] Vasheka OV, Bezsmertna OO (2012) Atlas of ferns of the flora of Ukraine. Palyvoda A.V., Kyiv

[CR63] Wolf PG, Rowe CA, Kinosian SP et al (2019) Worldwide relationships in the fern genus *Pteridium* (bracken) based on nuclear genome markers. Am J Bot 106:1365–1376. 10.1002/ajb2.1365 31545874 10.1002/ajb2.1365 PMC6856829

[CR64] World Flora Online. https://wfoplantlist.org. Accessed 24 January 2024

[CR65] Zając A, Zając M, Afranowicz-Cieślak R, et al (eds) (2019) Atlas rozmieszczenia roślin naczyniowych w Polsce: dodatek. Instytut Botaniki Uniwersytetu Jagiellońskiego, Kraków

[CR66] Zenkteler E, Nowak O (2019) Application of morphometric study to discriminate *Pteridium aquilinum* (L.) Kuhn subsp. *pinetorum* (C.N. Page & R.R. Mill 1995) J.A. Thomson in Poland. Biod Res Cons 56:1–12. 10.2478/biorc-2019-0015 10.2478/biorc-2019-0015

[CR67] Zenkteler E, Michalak KM, Nowak O (2022) Characteristics of indusia and sori in the two subspecies of *Pteridium aquilinum* (L.) Kuhn. occurring in Poland. Biod Res Cons 67:1–8. 10.2478/biorc-2022-0010 10.2478/biorc-2022-0010

[CR68] Zhou S, Dong W, Chen X et al (2014) How many species of bracken (*Pteridium*) are there? Assessing the Chinese brackens using molecular evidence. Taxon 63:509–521. 10.12705/633.9 10.12705/633.9

