# Supplementary material for: A new insight on the genus Pteridium (Dennstaedtiaceae) in Europe based on a revision in the flora of Slovakia
Source: Bot Stud. 2024 Aug 14;65:24. doi: 10.1186/s40529-024-00423-1 (PMC11324619; doi:10.1186/s40529-024-00423-1)
Supplement: Supplementary file 1 — Supplementary Material 1. Known locations of Pteridium aquilinum (L.) Kuhn within Slovakia: a) P. aquilinum subsp. aquilinum, b) P. aquilinum subsp. pinetorum (C.N. Page & R.R. Mill) J.A. Thomson [file 40529_2024_423_MOESM1_ESM.docx]

Supplementary materials A

**Known locations of *Pteridium aquilinum* (L.) Kuhn within Slovakia:**

**A) *Pteridium aquilinum* subsp. *aquilinum***

**4. Záhorská nížina^[[1]](#footnote-1)^**

Záhorie, medzi Šaštínom a Lakšárskou Novou Vsou [Záhorie region, between Šaštín village and Lakšárska Nová Ves village], 5.VI.1948, leg. J. Futák, det. J. Záborský, *SLO* (*48.6103497ºN, 17.1741228ºE*^[[2]](#footnote-2)^)

**5. Devínska Kobyla**

Okres Devínska Kobyla: Bratislava – Patrónka [Devínska Kobyla phytogeographical district: Bratislava city – Patrónka city district], 12.X.1963, leg. E. Schidlay, *SAV* (*48.174915ºN, 17.080242ºE*)

Bratislava, na západ od žel. zastávky Červený Most [Bratislava city, west of the railway station Červený Most], ca 200 m, 23.VII.1959, leg. & det. Schidlay, *SAV* (*48.1699361ºN, 17.0737975ºE*)

**5. Devínska Kobyla / 6. Podunajská nížina**

v lese nad 8. Landmühle, hojne [in the forest above the 8^th^ mill, often (*probably Mlynská dolina valley in Bratislava city*^[[3]](#footnote-3)^)], IX.1887, leg. J. A. Bäumler, BP, #29804 (*48.1523669ºN, 17.0758039ºE*)

**8. Východoslovenská nížina**

župa Zemplín, Kráľovský Chlmec, in silver "Erős" [Zemplín county, Kráľovský Chlmec town, in the forest "Erős"], IX.1927, leg. A. Margittai, *BP*, #29821 (*48.4218469ºN, 21.9734317ºE*)

na vrchu "Zempléni Szijt", v lesoch vrchu "Hossúhegy" pri Nagy Toronya [on the "Zempléni Szijt" hill, in the forests on "Hossúhegy" hill near Veľká Tŕňa village], ca 250 m, 29.VIII.1941, leg. L. Timár, *BP*, #29773 (*48.4732636ºN, 21.6915208ºE*)

**9. Biele Karpaty (južná časť)**

Ivanovské seče [*maybe meadows near the Ivanovce village, SW from Trenčín town*], IX.1868, leg. J.L. Holuby, *BP*, #29807 (*48.8320342ºN, 17.8986628ºE*)

**10. Malé Karpaty**

Marienthaler Wald [the forest *probably near the Mariánka village close to the Bratislava city*], VII.1887, leg. J.A. Bäumler, *BP*, #29803 (*48.2489056ºN, 17.0698386ºE*)

V Limbašskej doline pri Pezinku [in Limbach valley near the Pezinok town], 19.08.1912, leg. J.L. Holuby, *BRA* (*48.2751969ºN, 17.2516389ºE*)

**14b. Vtáčnik**

Prochotská dolina pod Vtáčnikom [Prochoť valley below the Mt. Vtáčnik], 10.IX.1907, leg. J. Tuzson, *BP*, #49181 (*48.6221806ºN, 18.6899786ºE*)

**14e. Štiavnické vrchy**

Prenčov, vrch Sitno [Prenčov village, Mt. Sitno], 20.X.1891, leg. A. Kmeť, *BP*, #29806 (*48.4030750ºN, 18.8775617ºE*)

Banská Štiavnica, Sitno, farská lúka [Banská Štaivnica town, Mt. Sitno, the pastor meadow], VI.1900, leg. S. Jávorka, *BP*, #29799 (*48.4022700ºN, 18.8764994ºE*)

**14f. Javorie**

Banská Bystrica Region, Lučenec District, near Divín village, close to Divínske Lazy, 48.483611ºN, 19.470556ºE, 03.09.2021, Observation by peternociar: *https://www.inaturalist.org/observations/93485143*

**20. Vihorlatské vrchy**

Prešov Region, Snina District, near Snina town, 48.977383ºN, 22.183333ºE, 17.07.2020, Observation by ivoholy: *https://www.inaturalist.org/observations/53364249*

**21a. Malá Fatra (Lúčanská Fatra)**

Martinské hole, v lese, Stráne [Martinské hole Mts, in the forest, Stráne (*perhaps today's part of Martin town on the western edge of the town*)], 09.07.1961, leg. & det. F. Dočolomanský, *BRA*, # 15 (*49.0819908ºN, 18.8975708ºE*)

**21c. Veľká Fatra**

v lesoch *Pinus sylvestris*, Laskomerská dolina pri Banskej Bystrici [in the *Pinus sylvestris* forests in Laskomerská dolina valley near the Banská Bystrica town], 25.VIII.1865, leg. A. Márkus, *BP*, #29809 (*48.7476092ºN, 19.1228658ºE*)

**21d. Chočské vrchy**

Chočské vrchy, Lúčky, vápenec [Chočské vrchy Mts, Lúčky village, the limestone], 2.VIII.1874, leg. Bohatsch, *BP*, #29803 (*49.1322167ºN, 19.4006997ºE*)

**24. Pieniny**

Pieniny, Golica [Pieniny Mts, Mt. Golica], 26.VIII.1953, leg. J. Májovský, det. J. Záborský, *SLO* (*49.4080911ºN, 20.4461603ºE*)

**27a. Biele Karpaty (severná časť)**

okres Trenčín, Horná Súča, boro-bukový les [Trenčín district, Horná Súča village, pine-beach forest], 450 m, 2.IX.1956, leg. & det. Schidlay, *SAV* (*48.9737661ºN, 17.9729342ºE*)

**B) *Pteridium aquilinum* subsp. *pinetorum* (C.N. Page & R.R. Mill) J.A. Thomson**

**2. Ipeľsko-rimavská brázda**

Šiatoroš, S od Fiľakova, na kopci "Tri chotáre" v listantom lese [Šiatoroš village, north of the Fiľakovo town, on the "Tri chotáre" hill in the deciduous forest], ca 300 m, 3.X.1959, leg. & det. Schidlay, *SAV*; Rev.: *P. pinetorum*, 12.10.2022, M. Peregrym (*48.1935592ºN, 19.8023744ºE*)

Rimavská Sobota, … *unreadable* *text*…, 28.5.1866, leg. J. Fábry, *BRA*, # 12; Rev.: *P. pinetorum*, 12.10.2022, M. Peregrym (*48.380999ºN, 20.022595ºE*)

**4. Záhorská nížina**

Záhorie: Lozorno, borové lesy na S od obce [Záhorie region, Lozorno village, pine forests north the village], alt. 180 m, 7.VIII.1951, leg. M. Ružička, det. E. Schidlay, *SAV*; Rev.: *P. pinetorum*, 12.10.2022, M. Peregrym (*48.351713ºN, 17.047338ºE*)

Záhorie, bahno pri Lakšárskej Novej Vsi [Záhorie region, peat bog near Lakšárska Nová Ves village], ca 167 m, 25.6.1963, leg. & det. E. Bosáčková, *BRA*, # 26; Rev.: *P. pinetorum*, 12.10.2022, M. Peregrym (*48.5844442ºN, 17.1770947ºE*)

Záhorie – Lakšánska Nová Ves, borina neďaleko starého židovského cintorina [Záhorie region, Lakšárska Nová Ves village, pine forest close to the old Jewish cemetery], alt. 180 m, 7.IV.1972, leg. & det. M. Manica, *ZV*, #16528, #16529, #16530, #16531, #16532, #16533, Rev.: *P. pinetorum*, 11.11.2022, M. Peregrym (*48.578931ºN, 17.183465ºE*)

Kúty: in silvis prope pag. Šajdik Humenec [Kúty town, in the forest near Šajdíkové Humence village], VIII.1935, leg. Ferd. Weber, *BRA*, #40, Rev.: *P. pinetorum*, 12.10.2022, M. Peregrym (*48.651842ºN, 17.274198ºE*)

Záhorská nížina, polesie Šajdíkové Humence, viate piesky, borovicové lesy [Záhorská nížina region, Šajdíkové Humence forestry, blowing sands, pine forests], 11.8.1965, leg. A. Bottlíková-Bergerová, *BRA*; Rev.: *P. pinetorum*, 12.10.2022, M. Peregrym (*48.6535669ºN, 17.2685903ºE*)

**5. Devínska Kobyla**

Lamač, Zigeunergraben [Lamač (*today district of the Bratislava city*), Gypsy Graben], 1.VII.1900, leg. C. Mergl, *SAV*; Rev.: *P. pinetorum*, 12.10.2022, M. Peregrym (*48.2000011ºN, 17.0572322ºE*)

Malé Karpaty, Lamač, južne od obce, kóta 262,2 m, medzi Líščím údolím a hradskou [Malé Karpaty Mts, Lamač (*today district of the Bratislava city*), south of the village, elevation 262,2 m, between Líščie údolie valley and road], *Querceto-Carpinetum*, 18.VII.1962, leg. Krippelová, Hubová, det. Krippelová, *SAV*; Rev.: *P. pinetorum*, 12.10.2022, M. Peregrym (*48.1717083ºN, 17.0545994ºE*)

**6. Podunajská nížina**

Čistiny, les [Čistiny locality, forest], 12.VII.1968, leg. & det. D. Hollá, *SLO*, Rev.: *P. pinetorum*, 12.10.2022, M. Peregrym (*47.854144ºN, 18.624516ºE*)

**7. Košická kotlina**

near Košice city, Lorinčík area, 48.693316ºN, 21.188616ºE, 17.05.2019, Observation by viktria3: *https://www.inaturalist.org/observations/25251877*

**8. Východoslovenská nížina**

(Východné Slovensko), upevnené duny južne od Beši: okolo horárne, v kroviskách [Eastern Slovakia, fixed dunes south of the Beša village, around of the forest house, in the bushes], 22.V.1963, leg. J. Májovský, *SLO*; Rev.: *P. pinetorum*, 12.10.2022, M. Peregrym (*48.5232514ºN, 21.9445783ºE*)

Potiská nížina, Ižkovce [Potiská nížina region, Ižkovce village]*, Querceto-Carpinetum*, ca 103 m, 17.III.1960, leg. & det. Schidlay, *SAV*; Rev.: *P. pinetorum*, 12.10.2022, M. Peregrym (*48.5579219ºN, 21.9552836ºE*)

**9. Biele Karpaty (južná časť)**

Zemianske Podhradie [Zemianske Podhradie village], 1865, leg. J.L. Holuby, *BRA*, # 13; Rev.: *P. pinetorum*, 12.10.2022, M. Peregrym (*48.8345061ºN, 17.8356631ºE*)

**10. Malé Karpaty**

Bratislava Region, Pezinok District, near Limbach village, 48.317066ºN, 17.206962ºE, 13.08.2023, Observation by vladislav_marusic: *https://www.inaturalist.org/observations/178504433*

pramenisko nad Hor. Rasuchovom, sev. Trstína [spring above the Horný Rasuchov north of the Trstín village], 300 m, 4.VII.1960, leg. & det. Krippelová, Jasičová, *SAV*; Rev.: *P. pinetorum*, 12.10.2022, *M. Peregrym* (*48.5411164ºN, 17.4696231ºE*)

**11. Považský Inovec**

Dubodiel, in Fageto [Dubodiel village, in beech forest], *BRA*, # 86; Rev.: *P. pinetorum*, 12.10.2022, M. Peregrym (*48.7626542ºN, 18.1061261ºE*)

Dubodiel [Dubodiel village], *collector and data are unknown, SAV*, Rev.: *P. pinetorum*, 12.10.2022, M. Peregrym (*48.7616706ºN, 18.0989128ºE*)

Jastrabie, v doline Svinica [Jastrabie village, in the Svinica valley], ca 340 m, 26.X.1961, leg. & det. Schidlay, *SAV*; Rev.: *P. pinetorum*, 12.10.2022, M. Peregrym (*48.7851800ºN, 18.1015942ºE*)

Inovecké pohorie, Mníchova Lehota, Krásnová dolina [Inovecké pohorie Mts, Mníchova Lehota village, Krásnova dolina valley], 370 m, 12.7.1956, leg. Schidlay, *SAV*; Rev.: *P. pinetorum*, 12.10.2022, M. Peregrym (*48.8092028ºN, 18.0849222ºE*)

**12. Tríbeč**

Nitra Region, Zlaté Moravce District, near Skýcov village, 48.484363ºN, 18.416336ºE, 21.09.2022, Observation by lucy563: *https://www.inaturalist.org/observations/135890772*

Vrez pri Krnči (Tríbeč. pohorie) [Vrez hill near Krnča village, Tríbečské pohorie Mts], ca 350 m, 24.VII.1972, leg. & det. Kováčiková, *SLO*; Rev.: *P. pinetorum*, 12.10.2022, M. Peregrym (*48.5454169ºN, 18.2923322ºE*)

**13. Strážovské a Súľovské vrchy**

Prievidza, na svahoch vrchu Vysoký Rokoš nad dedinou Vrbany, vápencový substrát [Prievidza town, on the slope of the Mt. Vysoký Rokoš above the Vrbany village, limestone substrate], 800 m, 9.7.1939, leg. V. Valenta, *BRA*, # 75; Rev.: *P. pinetorum*, 12.10.2022, M. Peregrym (*48.7712675ºN, 18.4339136ºE*)

Strážovské vrchy, Soblahov, kraj bývalej železničnej dráhy nad dedinou [Strážovské vrchy Mts, Soblahov village, the margin of the former railway station above the village], 31.VII.1927, leg. & det. Schidlay, *SAV*; Rev.: *P. pinetorum*, 12.10.2022, M. Peregrym (*48.8542244ºN, 18.0671869ºE*)

Okres Trenčín, Soblahov, hojne v mladej machnatej bučine [Trenčín district, Soblahov village, abundantly in the young mossy beech forest], 350 m, 22.X.1956, leg. & det. Schidlay, *SAV*; Rev.: *P. pinetorum*, 12.10.2022, M. Peregrym (*48.8655128ºN, 18.1017558ºE*)

Okres Trenčín, Soblahov, južný svah [Trenčín district, Soblahov village, south slope], 350 m, 1.VIII.1956, leg. & det. Schidlay, *SAV*; Rev.: *P. pinetorum*, 12.10.2022, M. Peregrym (*48.8701425ºN, 18.0947178ºE*)

Strážovská hornatina, Baba k. 669 m [Strážovská hornatina Mts, Baba hill, elevation 669 m], 22.V.1966, leg. M. Kaleta, *BRA*, #55, Rev.: *P. pinetorum*, 12.10.2022, M. Peregrym (*48.915806ºN, 18.499034ºE*)

Omšenie, Baba, západný svah, čistinka v bučine [Omšenie village, west slope, open place in beech forest], ca 500 m, 10.X.1961, leg. & det. Schidlay, *SAV*; Rev.: *P. pinetorum*, 12.10.2022, M. Peregrym (*48.9111892ºN, 18.2340203ºE*)

Strážovská hornatina, horný okraj pasienku na úpätí Vápča, záp. exp. [Strážovská hornatina Mts, top margin of the pasture on the foot of the Mt. Vápeč, west exposition], 20.VI.1962, leg. K. Zahradníková, *SAV*; Rev.: *P. pinetorum*, 12.10.2022, M. Peregrym (*48.9356897ºN, 18.3171633ºE*)

Okres Ilava: Košecké Rovné [Ilava district, Košecké Rovné village], 28.X.1957, leg. E. Schidlay, *SAV*, Rev.: *P. pinetorum*, 12.10.2022, M. Peregrym (*48.960758ºN, 18.409923ºE*)

Strážovská hornatina, okres Ilava, vrch Sokol NNW od Hornej Lehoty, svetlá kriačina [Strážovská hornatina Mts, Ilava district, Mt. Sokol NNW Horná Lehota village, lighty bush], 16.VI.1963, leg. & det. J. Futák, *SAV*; Rev.: *P. pinetorum*, 12.10.2022, M. Peregrym (*48.9706911ºN, 18.2930450ºE*)

Strážovská hornatina, medzi Trstínom a Mojtínom, niže mlyna "Uhliská" na okraji bučiny [Strážovská hornatina Mts, between Trstín village and Mojtín village, in the margin of the beech forest below the "Uhliská" mill], 400 m, 21.VI.1956, leg. & det. Schidlay, *SAV*; Rev.: *P. pinetorum*, 12.10.2022, M. Peregrym (*49.0076711ºN, 18.4274933ºE*)

Trenčín Region, Považská Bystrica District, near Ďurďové village, 49.036243ºN, 18.500478ºE, 17.06.2019, Observation by vcircle: *https://www.inaturalist.org/observations/27150576*

Rajecké Teplice, Skalky, ca 550 m, vápenec [Rajecké Teplice village, Mt. Skalky, limestone], 16.8.1963, leg. & det. F. Dočolomanský, *BRA*, # 26; Rev.: *P. pinetorum*, 12.10.2022, M. Peregrym (*49.1419667ºN, 18.6849683ºE*)

Súľovské skaly [Súľovské skaly Mts], … *unreadable* *text*…, 450 m, 22.VI.1956, leg. & det. Schidlay, *SAV*; Rev.: *P. pinetorum*, 12.10.2022, M. Peregrym (*49.1767739ºN, 18.5859625ºE*)

Súľovské skaly [Súľovské skaly Mts], 23.VI.1942, leg. & det. F. Nábělek, *SAV*; Rev.: *P. pinetorum*, 12.10.2022, M. Peregrym (*49.1733878ºN, 18.5832836ºE*)

Súľovské skaly, kopec "Stavnica", severný svah [Súľovské skaly Mts, Mt. “Stavnica”, north slope], ca 390 m, 10.VII.1959, leg. & det. Schidlay, *SAV*; Rev.: *P. pinetorum*, 12.10.2022, M. Peregrym (*49.1590206ºN, 18.5713100ºE*)

Súľovské skaly [Súľovské skaly Mts], 23.VI.1942, leg. Nábělek, Futák, Brižický, det. J. Futák, *SLO*; Rev.: *P. pinetorum*, 12.10.2022, M. Peregrym (*49.1748539ºN, 18.5839419ºE*)

Súľovské skaly [Súľovské skaly Mts], 9.VI.1947, leg. J. Futák, det. J. Záborský, *SLO*; Rev.: *P. pinetorum*, 12.10.2022, M. Peregrym (*49.1735633ºN, 18.5826117ºE*)

**14b. Vtáčnik**

župa Bars, in silvis prope Bükköskút [Bars county, in the forest near Bükköskút (= Bukovina) village], VIII.1908, leg. J. Tuzson, BP, #49113; Rev.: *P. pinetorum*, 6.10.2022, M.Peregrym (48.537570ºN, 18.731130ºE)

**14c. Kremnické vrchy**

Kremnické pohorie – Kováčová, po porastoch na l’avej strane údolia [Kremnické pohorie Mts, Kováčová village, on the left side of the valley]. Alt. 350 – 400 m, 7.VI.1967, Leg. & Det. M. Manica, *ZV*, #15121, Rev.: *P. pinetorum*, 11.11.2022, M. Peregrym (*48.604297ºN, 19.086390ºE*)

Kremnické pohorie – Kováčová, lesy nad Kováčovou dolinou, na sever [Kremnické pohorie Mts, Kováčová village, forests above Kováčová valley, on the north]. Alt. 350 m, 8.VII.1969, Leg. & Det. M. Manica, *ZV*, #15066, Rev.: *P. pinetorum*, 11.11.2022, M. Peregrym (*48.616506ºN, 19.071101ºE*)

**14d. Poľana**

Masív Pol’any – Očová, smrekový les na SV od obce [Poľana Mts, Očová village, spruce forest north-east of the village]. Alt. 460 – 500 m, 1.VI.1971, leg. & det. M. Manica, *ZV*, #16729, #16730, Rev.: *P. pinetorum*, 11.11.2022, M. Peregrym (*48.616040ºN, 19.307187ºE*)

**14e. Štiavnické vrchy**

Krnišov [Krnišov village], VI.1876, leg. A. Kmeť, *BRA*, # 13; Rev.: *P. pinetorum*, 12.10.2022, M. Peregrym (*48.3540683ºN, 18.9741533ºE*)

Pri Dekýš [near Dekýš village], 20.10.1886, leg. A. Kmeť, *BRA*, # 13; Rev.: *P. pinetorum*, 12.10.2022, M. Peregrym (*48.3954244ºN, 18.8155061ºE*)

Hronská Breznica [Hronská Breznica village], 300 m, VI.35, leg. & det. Nábĕlek, *SAV*; Rev.: *P. pinetorum*, 12.10.2022, M. Peregrym (*48.565387ºN, 18.995591ºE*)

**14f. Javorie**

Javorie, Malý Lysec [Javorie Mts, Mt. Malý Lysec], 10.VIII.1971, leg. & det. Schwarzová, *SLO*; Rev.: *P. pinetorum*, 12.10.2022, M. Peregrym (*48.4953603ºN, 19.2417056ºE*)

SV od Modrého Kameňa, Príboj - Šula, okraj lúky [north-east of the Modrý Kameň town, Príboj – Šula locality, the margin of the meadow], 4.XII.1959, leg. & det. Schidlay, *SAV*; Rev.: *P. pinetorum*, 12.10.2022, M. Peregrym (*48.3414633ºN, 19.4106858ºE*)

**15. Slovenské rudohorie**

Ďubákovo – cesta hrebeňom na Diel, južná expoz. [Ďubákovo village, the road along the ridge to the Mt. Diel, south exposition], 900 m, 22.VIII.1973, leg. & det. Dvořáková, *SLO*; Rev.: *P. pinetorum*, 12.10.2022, M. Peregrym (*48.5665789ºN, 19.7839839ºE*)

Banská Bystrica Region, Poltár District, near Ďubákovo village, 48.547214ºN, 19.735833ºE, 31.10.2021, Observation by fero: *https://www.inaturalist.org/observations/100018961*

Vaľkovo a České Brezovo [Vaľkovo village and České Brezovo village], … *unreadable* *text*…, 4.8.1960, *collector unreadable*, *BRA*, # 28; Rev.: *P. pinetorum*, 12.10.2022, M. Peregrym (*48.4730644ºN, 19.8148864ºE*)

nad obcou Klenovec neďaleko lesovne "Čierny potok", na pastvinách vo veľkých skupinách [above the Klenovec town close to the forest house “Čierny potok”, on the pastures in large groups], ca 340 m, 23.IX.1960, leg. & det. T. Blattny, *SAV*; Rev.: *P. pinetorum*, 12.10.2022, M. Peregrym (*48.6028386ºN, 19.8809953ºE*)

Tót Hegymeg [Tót Hegymeg (*= Horné Zahorany*) village], VIII.1885, leg. A. Richter, *BP*, #29819; Rev.: *P. pinetorum*, 6.10.2022, M. Peregrym (*48.4614847ºN, 20.0148683ºE*)

Distr. Revúca, region carstica Jelšavský kras: Jugum Dúbrava supra opp. Jelšava [Revúca district, karst region Jelšavský kras, Dúbrava ridge above the Jelšava town], 450 m s.m., 10.VI.1954, leg. Hendrych, *SAV*; Rev.: *P. pinetorum*, 12.10.2022, M. Peregrym (*48.6614817ºN, 20.2512794ºE*)

Montes Slovenské Rudohorie in declivibus collium Ortás et Repisko, a pagi Ochtiná ad meridiem versus [Slovenské Rudohorie Mts, on the slopes of the Ortás and Repisko hills, from the Ochtiná village to the south], alt. c. 400 – 650 m, leg. V. Vašák, *BRA*, #134, Rev.: *P. pinetorum*, 12.10.2022, M. Peregrym (*48.674244ºN, 20.336355ºE*)

Slovenské Rudohorie, Stoličné vrchy, Vlachovo communitas, Stožok collis, loc. Na tábli, cesta do Brdárky [Slovenské Rudohorie phytogeographical region, Stolické vrchy Mts, Vlachovo village, Mt. Stožok, Na tábli locality, the road to the Brdárka village], alt. c. 650 m, *Querco-Carp.*, 4.VII.1955, leg. & det. Hajdúk, *BRA*, #258, Rev.: *P. pinetorum*, 12.10.2022, M. Peregrym (*48.771435ºN, 20.382182ºE*)

*unreadable text* (*probably Spiš region, Helcmanovce village*), VIII.1870, leg. M. Ruby, *BP*, #29800; Rev.: *P. pinetorum*, 6.10.2022, M. Peregrym (*48.8290694ºN, 20.8713914ºE*)

pri Gelnici, v lese na vrchu Wendi [near Gelnica town, in the forest on the Mt. Wendi], 11.VIII.1875, leg. L. Udránszky, *BP*, #29810; Rev.: *P. pinetorum*, 6.10.2022, M. Peregrym (*48.8585553ºN, 20.9503558ºE*)

**16. Muránska planina**

Tisovec, Slávča [Tisovec town, Slávča valley], 7.6.1923, leg. V. Vraný, *BRA*, # 22; Rev.: *P. pinetorum*, 12.10.2022, M. Peregrym (*48.7102925ºN, 19.9439114ºE*)

Muráň, cestou na Klin [Muráň village, along the way to the Klin locality], 5.7.1962, leg. & det. F. Dočolomanský, *BRA*, # 21; Rev.: *P. pinetorum*, 12.10.2022, M. Peregrym (*48.7489039ºN, 20.0312442ºE*)

Muráňska plošina – na sever od obce Muraň, bočný hrebeň od juhu na Poludnicu [Muránska planina Mts, north of the Muráň village, side ridge from south to the Poludnica locality], alt. 700 – 800 m, 23.VI.1962, leg. & det. M. Manica, *ZV*, #15664, Rev.: *P. pinetorum*, 11.11.2022, M. Peregrym (*48.751085ºN, 20.038407ºE*)

Muránsky hrad [Muráň castle], 10.8.1946, *collector unreadable*, *BRA*, # 28; Rev.: *P. pinetorum*, 12.10.2022, M. Peregrym (*48.7586228ºN, 20.0591178ºE*)

**17. Slovenský raj**

Slovenský raj, Communitas: Vernár, Stratená, Collis: Popová, Flumen: Hnilec, pri ceste Poprad 100 m, SW, 30°, dolomit, rendzina [Slovenský raj Mts, villages: Vernár, Stratená, hill: Popová, river: Hnilec, next to the road Poprad 100 m, south-west, 30°, dolomite, rendzina], *Calamagrostietum variae*, ca 1050 m, 8.8.1956, leg. & det. Hajdúk, *BRA*, # 258; Rev.: *P. pinetorum*, 12.10.2022, M. Peregrym (*48.8961839ºN, 20.2471192ºE*)

Slovenský raj, Communitas: Vernár, Stratená, Collis: Popová, Flumen: Hnilec, S, 30°, dolomit, rendzina [Slovenský raj Mts, villages: Vernár, Stratená, hill: Popová, river: Hnilec, south, 30°, dolomite, rendzina], *Calamagrostietum variae*, ca 1100 m, 2.8.1956, leg. & det. Hajdúk, *BRA*, # 258; Rev.: *P. pinetorum*, 12.10.2022, M. Peregrym (*48.8961839ºN, 20.2471192ºE*)

Slovenský raj, Spišské Tomášovce, Biely potok, Hornád, roklina Kyseľ, S (N), 20°, kalcit, rendzina [Slovenský raj Mts, Spišské Tomášovce village, Biely Potok stream, Hornád river, Kyseľ gorge, south (north), calcite, rendzina], *Piceetum calcicolum*, ca 600 m, 1956, leg. & det. Hajdúk, *BRA*, # 259; Rev.: *P. pinetorum*, 12.10.2022, M. Peregrym (*48.890851*ºN*, 20.241658ºE*)

Slovenský raj, Spišské Tomášovce, Tomášovská Belá - Hornád, Klauzy, N (S), 20°, kalcit [Slovenský raj Mts, Spišské Tomášovce village, Tomášovská Belá – Hornád river, Klauzy locality, north (south), 20°, calcite], ca 900 m, 1956, leg. & det. Hajdúk, *BRA*, # 259; Rev.: *P. pinetorum*, 12.10.2022, M. Peregrym (*48.9113322ºN, 20.4182225ºE*)

*unreadable* *text (probably* Spišská Nová Ves town*)*, VIII.1893, leg. Márton, *BRA*, # 13; Rev.: *P. pinetorum*, 12.10.2022, M. Peregrym (*48.9404386ºN, 20.5507986ºE*)

**18. stredné Pohordnádie**

medzi Ťahanovcami a Košickou Novou Vsou, údolie potoka Nolnok, v jelšine pospolite [between Ťahanovce and Košická Nová Ves villages, the Nolnok creek valley, in alder forest in the groups], 260 m, 13.III.1960, leg. & det. Schidlay, *SAV*; Rev.: *P. pinetorum*, 12.10.2022, M. Peregrym (*48.7481103ºN, 21.2762139ºE*)

near Košice city, Skalky area, 48.768173ºN, 21.275093ºE, 22.04.2020, Observation by martin: *https://www.inaturalist.org/observations/42828167*

Košické Hámre, na vrchu "Braseler", župa Abov [Košické Hámre village, on the “Braseler” hill, Abov county], 1.IX.1918, leg. Hulják, *BP*, #29411; Rev.: *P. pinetorum*, 6.10.2022, M. Peregrym (*48.8286144ºN, 21.0817061ºE*)

dolina Hornádu, Ružín [the Hornád river valley, Ružín village], 4.IX.1946, leg. & det. J. Futák, *SLO*; Rev.: *P. pinetorum*, 12.10.2022, M. Peregrym (*48.8730803ºN, 21.0458236ºE*)

**19. Slanské vrchy**

Slanské vrchy, nad Kokošovcami [Slanské vrchy Mts, above Kokošovce village], 27.VI.1955, leg. & det. Schidlay, *SAV*; Rev.: *P. pinetorum*, 12.10.2022, M. Peregrym (*48.9455539ºN, 21.3536125ºE*)

Slánske vrchy, Podhradík, na pasienku pospolite na okraji krovín [Slanské vrchy Mts, Podhradík village, on the pasture in groups on the margin of the bushes], ca 500 m, 8.IX.1955, leg. & det. Schidlay, *SAV*; Rev.: *P. pinetorum*, 12.10.2022, M. Peregrym (*48.9998244ºN, 21.3512200ºE*)

Kapušianske kopce, Lysá stráž [Kapušianske kopce hills, Mt. Lysá stráž], 21.VI.1947, leg. & det. Májovský, *SLO*, Rev.: *P. pinetorum*, 12.10.2022, M. Peregrym (*49.083817ºN, 21.230051ºE*)

**20. Vihorlatské vrchy**

(východné Slovensko), kóta Dupna - Chlmec, bučiny, exp. východ [eastern Slovakia, elevation Dupna – Chlmec, beech forests, east orientation], 5.VI.1975, leg. & det. Hudáková, *SLO*; Rev.: *P. pinetorum*, 12.10.2022, M. Peregrym (*48.9010883ºN, 21.9189283ºE*)

kóta Sokol - Tretia skala, hrebeň, exp. S – Z [elevation Sokol – Tretia skala (*probably Mt. Humenský Sokol*), the ridge, orientation south-west], 20.VIII.1975, leg. & det. Hudáková, *SLO*, (*48.9057122ºN, 21.9242078ºE*)

na pasienkoch pod Sninským kameňom pri chodníku ku Sninským jazerám [on pastures below the Mt. Sninský kameň near the path to the Sninské jazerá lakes], 10.VII.1967, leg. & det. J. Májovský, *SLO*; Rev.: *P. pinetorum*, 12.10.2022, M. Peregrym (*48.9067997ºN, 22.2029072ºE*)

Ung megye viránya, Prikopa, a szőlők alatti cserjésben [Uh county, Prikopa (*= correctly Priekopa*) village, in the bush under the vine], 2.IX.1883, leg. Dietz Sándor, *BP*, #29580, (*48.752575ºN, 22.269503ºE*)

južný svah Vihorlatu nad obcou Koromľa [south slope of the Mts Vihorlat above the Koromľa village], 17.IX.1949, leg. & det. J. Michalko, *SLO*; Rev.: *P. pinetorum*, 12.10.2022, M. Peregrym (*48.7251519ºN, 22.2987800ºE*)

**21a. Malá Fatra (Lúčanská Fatra)**

Trenčín Region, Prievidza District, near Kľačno village, 48.940416ºN, 18.69915ºE, 5.10.2022, Observation by katarinakyselova: *https://www.inaturalist.org/observations/137727161*

Lúčanská Malá Fatra, Vrútky – Háj [Lúčanská Malá Fatra Mts, Vrútky town, Háj locality], 10.7.1977, leg. & det. J. Medovič, *BRA*, # 119; Rev.: *P. pinetorum*, 12.10.2022, M. Peregrym (*49.1072400ºN, 18.9185136ºE*)

**21b. Malá Fatra (Krivánská Fatra)**

Turčianska kotlina, Lipovec, pri Váhu [Turčianska kotlina basin, Lipovec village, near Váh river], 23.7.1977, leg. & det. J. Medovič, *BRA*, # 119; Rev.: *P. pinetorum*, 12.10.2022, M. Peregrym (*49.1254981ºN, 18.9256375ºE*)

Žilina Region, Martin District, near Turčianske Kľačany village, 49.136111ºN, 18.953611ºE, 29.08.2022, Observation by mykytaperegrym: *https://www.inaturalist.org/observations/151576005*

Malá Fatra, Vrátna dolina [Malá Fatra Mts, Vrátna dolina valley], 10.VI.1947, leg. & det. J. Futák, *SLO*; Rev.: *P. pinetorum*, 12.10.2022, M. Peregrym (*49.2360342ºN, 19.0395742ºE*)

Žilina Region, Dolný Kubín District, near Kraľovany village, 49.152282ºN, 19.10264ºE, 21.08.2023, Observation by merlin3003: *https://www.inaturalist.org/observations/179710246*

Chočské pohorie, Kraľovany, pravý breh Oravy povyše horárne Bystrička [Chočské pohorie Mts, Kraľovany village, right bank of the Orava river above the Bystrička forest house], ca 450 m, 3.IX.1973, leg. O. Hubová, C. Blatný, det. Hubová, *SAV*; Rev.: *P. pinetorum*, 12.10.2022, M. Peregrym (*49.1754078ºN, 19.1352822ºE*)

medzi Kraľovanmi a Párnicou, Bystrička, na južnom krovinatom svahu pri hradskej [between Kraľovany and Párnica villages, Bystrička valley, on the south shrubs slope above the road], 450 m, 9.VIII.1956, leg. & det. Schidlay, *SAV*; Rev.: *P. pinetorum*, 12.10.2022, M. Peregrym (*49.1763869ºN, 19.1324208ºE*)

**21c. Veľká Fatra**

near Banská Bystrica city, 48.749583ºN, 19.092672ºE, 14.05.2022, Observation by dana566: *https://www.inaturalist.org/observations/122800264*

near Banská Bystrica city, 48.749376ºN, 19.09223ºE, 14.05.2022, Observation by robertxd: *https://www.inaturalist.org/observations/116944319*

near Banská Bystrica city, 48.7475ºN, 19.095833ºE, 22.09.2022, Observation by mykytaperegrym: *https://www.inaturalist.org/observations/142737706*

boriny nad Riečkou a dolina B.B. vodovodov [pine forests above the Riečka village and valley of the Banská Bystrica´s ducts], 29.VI.1952, leg. Májovský, Bertová, Michalko, *SLO*; Rev.: *P. pinetorum*, 12.10.2022, M. Peregrym (*48.7639925ºN, 19.0739064ºE*)

Gader [Gader valley], 18.VII.1938, leg. & det. Dohnany, *SLO*; Rev.: *P. pinetorum*, 12.10.2022, M. Peregrym (*48.9495019ºN, 18.9602667ºE*)

Veľká Fatra, v Gaderskej doline [Veľká Fatra Mts, in the Gaderská dolina valley], 28.VII.1911, leg. Hulják, *BP*, # 29410; Rev.: *P. pinetorum*, 6.10.2022, M. Peregrym (*48.9505700ºN, 18.9659797ºE*)

Veľká Fatra, Gaderská dolina, Kozia skala [Veľká Fatra Mts, Gaderská dolina valley, Mt. Kozia skala], VIII.1908, leg. Hulják, BP, #29820; Rev.: *P. pinetorum*, 6.10.2022, M. Peregrym (*48.9604331ºN, 18.9940464ºE*)

Veľká Fatra, Necpalská dolina, smerom ku Kozej skale, na lesnej čistinke [Veľká Fatra Mts, Necpalská dolina valley, toward the Mt. Kozia skala, in the open place in the forest], ca 800 m, 22.VII.1975, leg. & det. Kováčiková, *SAV*; Rev.: *P. pinetorum*, 12.10.2022, M. Peregrym (*48.9668208ºN, 18.9999089ºE*)

Veľká Fatra: pri Krkavej skale nad Vlkolíncom, suché stráne, J exp. [Veľká Fatra Mts, near Mt. Krkavá skala above the Vlkolínec village, dry slopes, south exposition], 10º, Alt. 880 m, 20.VII.1974, leg. & det. L. Mucina, *BRA*, #114, Rev.: *P. pinetorum*, 12.10.2022, M. Peregrym (*49.046598ºN, 19.284845ºE*)

Žilina Region, near Ružomberok town, 49.064889ºN, 19.273668ºE, 19.08.2022, Observation by fero: https://*www.inaturalist.org/observations/131549214*

medzi Kraľovanmi a Rojkovom na severnom svahu vrchu Kopa [between Kraľovany and Rojkov villages on the north slope of the Mt. Kopa], 550 m, 15.VIII.1956, leg. & det. Schidlay, *SAV*; Rev.: *P. pinetorum*, 12.10.2022, M. Peregrym (*49.1468639ºN, 19.1455744ºE*)

**21d. Chočské vrchy**

Kraľovany, breh Oravy pri ceste [Kraľovany village, the bank of Orava river next the road], 17.6.1978, det. Berta, *BRA*, # 156; Rev.: *P. pinetorum*, 12.10.2022, M. Peregrym (*49.1550617ºN, 19.1388839ºE*)

Žilina Region, Ružomberok District, near Švošov village, 49.130692ºN, 19.20378ºE, 14.06.2015, Observation by fero: *https://www.inaturalist.org/observations/5284845*

Žilina Region, Ružomberok District, near Švošov village, 49.131213ºN, 19.20195ºE, 28.07.2020, Observation by fero*: https://www.inaturalist.org/observations/52683821*

Žilina Region, Ružomberok District, near Švošov village, 49.13726ºN, 19.198698ºE, 28.07.2020, Observation by fero: *https://www.inaturalist.org/observations/52683795*

Žilina Region, Ružomberok District, near Švošov village, 49.125738ºN, 19.185721ºE, 28.07.2020, Observation by fero: *https://www.inaturalist.org/observations/52683731*

Žilina Region, Ružomberok District, near Švošov village, 49.122015ºN, 19.185945ºE, 28.07.2020, Observation by fero: *https://www.inaturalist.org/observations/52683682*

Žilina Region, Ružomberok District, near Švošov village, 49.125406ºN, 19.191317ºE, 28.07.2020, Observation by fero: *https://www.inaturalist.org/observations/52683660*

Žilina Region, Ružomberok District, near Švošov village, 49.129018ºN, 19.206543ºE, 28.07.2020, Observation by fero: *https://www.inaturalist.org/observations/50649351*

Ružomberok, Čebrať [Ružomberok town, Mt. Čebrať], VII.1964, leg. V. Hodoval, det. Berta, *BRA*, # 156; Rev.: *P. pinetorum*, 12.10.2022, M. Peregrym (*49.1016772ºN, 19.2789597ºE*)

Žilina Region, Ružomberok District, near Valaská Dubová village, 49.143404ºN, 19.300599ºE, 05.09.2021, Observation by fero: *https://www.inaturalist.org/observations/93921870*

nad Valaskou Dubovou, pod Chočom, vlhké pramenisko [above Valaská Dubová village, below the Mt. Choč, the damp spring], 8.VII.1940, leg. & det. Dohnany, *SLO*; Rev.: *P. pinetorum*, 12.10.2022, M. Peregrym (*49.1456669ºN, 19.3058211ºE*)

Chočské pohorie, Bukov [Chočské pohorie Mts, Mt. Bukov], 4.6.1969, leg. Magdolenová, det. Paclová, *BRA*, #85; Rev.: *P. pinetorum*, 12.10.2022, M. Peregrym (*49.1469778ºN, 19.3613142ºE*)

**22. Nízke Tatry**

Nízke Tatry, Siná, dolina Kamenica, blízko potoka [Nízke Tatry Mts, Kamenica valley, close to the creek], ca 850 m, 28.VIII.1955, leg. & det. Schidlay, *SAV*; Rev.: *P. pinetorum*, 12.10.2022, M. Peregrym (*48.9962064ºN, 19.5818953ºE*)

Demänovská dolina, na pasienku spolu s *Potentilla tormentilla*, *Deschampsia*, na hlinitopiesočnatej tmavohnedej pôde [Demänovská dolina valley, on pasture together with *Potentilla tormentilla*, *Deschampsia*, on the loamy-sandy dark brown soil], 800 m, 28.VIII.1955, leg. & det. Schidlay, *SAV*; Rev.: *P. pinetorum*, 12.10.2022, M. Peregrym (*48.9827453ºN, 19.5951561ºE*)

Demänovská dolina, na výslnnom pasienku, neďaleko cesty [Demänovská dolina valley, on the sunny pasture, close to the road], cca 800 m, 28.VIII.1955, leg. & det. Schidlay, *SAV*; Rev.: *P. pinetorum*, 12.10.2022, M. Peregrym (*49.0062575ºN, 19.5826356ºE*)

Demänovská dolina, dolný koniec Demänovskej doliny [Demänovská dolina valley, the nether end of the valley], ca 800 m, 28.VIII.1955, leg. & det. Schidlay, *SAV*; Rev.: *P. pinetorum*, 12.10.2022, M. Peregrym (*49.0062575ºN, 19.5826356ºE*)

Demänovská dolina, Bohuš (pohorelisko) v Callunetu [Demänovská dolina valley, burnt-out place Bohuš, in *Callunetum*], 15.IX.1955, leg. A. Ščepka, det. K. Zahradníková, *SAV*; Rev.: *P. pinetorum*, 12.10.2022, M. Peregrym (*48.9870122ºN, 19.5695464ºE*)

Demänovská dolina [Demänovská dolina valley], ca 800 m, 28.VIII.1955, leg. & det. Schidlay, *SAV*; Rev.: *P. pinetorum*, 12.10.2022, M. Peregrym (*49.0124086ºN, 19.5842808ºE*)

Nízke Tatry, severná vápencová obruba, vrch Zapač, východný hrebeň [Nízke Tatry Mts, the north limestone fringe, Mt. Zapač, the east ridge], 750 m, 26.7.1956, leg. & det. J. Horníčková, *BRA*, # 126; Rev.: *P. pinetorum*, 12.10.2022, M. Peregrym (*49.0294422ºN, 19.7221464ºE*)

Nízke Tatry, vrch Zapač [Nízke Tatry Mts, Mt. Zapač], 700 m, 12.7.1968, leg. & det. J. Horníčková, *BRA*, # 126; Rev.: *P. pinetorum*, 12.10.2022, M. Peregrym (*49.0295267ºN, 19.7278111ºE*)

Nízke Tatry, Turková [Nízke Tatry Mts, Mt. Turková], Čierny Váh, 900 m, 4.VII.1974, leg. & det. E. Vartíková, *SLO*; Rev.: *P. pinetorum*, 12.10.2022, M. Peregrym (*49.0114392ºN, 19.9171931ºE*)

Comit. Gömör: in m. Popova ad Pusztamező [Gemer county, on the Mt. Popová above the Pusté Pole locality], 1.VIII.1932, (*unknown date and collector*), *BP*, #29366, (*48.8981067ºN, 20.2407753ºE*)

**22. Nízke Tatry / 26b. Spišské kotliny**

Baba [Mt. Baba], 6.9.1932, leg. G. Grodkovszky, *BRA*, # 47; Rev.: *P. pinetorum*, 12.10.2022, M. Peregrym (*49.0511181ºN, 20.2161450ºE*)

**23b. Vysoké Tatry**

Prešov Region, Poprad District, near Vysoké Tatry town, 49.14079ºN, 20.22629ºE, 9.08.2023, Observation by jiriklimes: *https://www.inaturalist.org/observations/177479603*

Alsó Tátrafűred [Alsó Tátrafűred (*= Dolný Smokovec*) village], 15.VIII.1888, leg. Desakó, *BP*, #29818; Rev.: *P. pinetorum*, 6.10.2022, M. Peregrym (*49.1298381ºN, 20.2441650ºE*)

**24. Pieniny**

pri ceste z Červeného Kláštora do Lechnica [near the road from the Červený Kláštor village to the Lechnica village], 11.VII.1968, leg. & det. J. Májovský, *SLO*; Rev.: *P. pinetorum*, 12.10.2022, M. Peregrym (*49.3977739ºN, 20.4168133ºE*)

Prielom Dunajca a Huta [the breakthrough of the Dunajec river and Huta creek], 8.8.1971, leg. Garajová, Bielunková, Hubová, *SAV*; Rev.: *P. pinetorum*, 12.10.2022, M. Peregrym (*49.4035253ºN, 20.4350453ºE*)

**25. Turčianska kotlina**

Žilina Region, Turčianske Teplice District, near Rakša village, 48.87787ºN, 18.891305ºE, 15.07.2023, Observation by hemala_vladimir: *https://www.inaturalist.org/observations/175259745*

**26a. Liptovská kotlina / 22. Nízke Tatry**

Žilina Region, Ružomberok District, near Ludrová village, 49.046013ºN, 19.339803ºE, 01.06.2020, Observation by fero: *https://www.inaturalist.org/observations/48891208*

**26b. Spišské kotliny**

Košice Region, Spišská Nová Ves District, near Spišské Tomášovce village, 48.946685ºN, 20.460717ºE, 17.09.2022, Observation by fero: *https://www.inaturalist.org/observations/136461007*

(Flora Tatra Magnae), in silvis frondosis ad Lengvart pr. Levoča [in deciduous forest near Lengvart (*= Dlhé Stráže*) village near Levoča town], IX.1915, leg. V. Greschik, *SLO*; Rev.: *P. pinetorum*, 12.10.2022, M. Peregrym (*49.0322506ºN, 20.5196814ºE*)

Levoča, in silvis frondosis ad Lengvárt [Levoča town, in deciduous forest near Lengvart (*= Dlhé Stráže*) village], IX.1915, leg. V. Greschik, *SLO*; Rev.: *P. pinetorum*, 12.10.2022, M. Peregrym (*49.0322506ºN, 20.5196814ºE*)

k jelšine pri kúpeľoch Lučivná [to the alder forest near Lučivná spa], 780 m, 10.VIII.1895, leg. *unreadable name*, *BP*, #29811; Rev.: *P. pinetorum*, 6.10.2022, M. Peregrym (*49.0509717ºN, 20.1372817ºE*)

**27a. Biele Karpaty (severná časť)**

Okres Trenčín, Horná Súča, dolina Bukovinka, na okraji listnatého krovia [Trenčín district, Horná Súča village, Bukovinka valley, in the margin of the deciduous forest], 400 m, 2.IX.1956, leg. & det. Schidlay, *SAV*; Rev.: *P. pinetorum*, 12.10.2022, M. Peregrym (*48.9686750ºN, 17.9752844ºE*)

okres Trenčín, Horná Súča, na okraji hrabového krovia [Trenčín district, Horná Súča village, in the margin of the hornbeam forest], 400 m, 2.IX.1956, leg. & det. Schidlay, *SAV*; Rev.: *P. pinetorum*, 12.10.2022, M. Peregrym (*48.9835558ºN, 17.9734922ºE*)

okres Trenčín, Horná Súča, boro-bukový les krovia [Trenčín district, Horná Súča village, the pine-beech forest], 450 m, 2.IX.1956, leg. & det. Schidlay, *SAV*; Rev.: *P. pinetorum*, 12.10.2022, M. Peregrym (*48.9737661ºN, 17.9729342ºE*)

Biele Karpaty, Horné Sŕnie, okraj lesa na lúke nad "Rajkovcom" [Biele Karpaty Mts, Horné Sŕnie village, the forest margin on the meadow above “Rajkovec”, 23.VII.1928, leg. & det. Schidlay, *SAV*; Rev.: *P. pinetorum*, 12.10.2022, M. Peregrym (*49.0187950ºN, 18.0758250ºE*)

**28. Západné Beskydy**

Žilina Region, Čadca District, near Čierne village, 49.517204ºN, 18.850889ºE, 20.07.2023, Observation by svatka: *https://www.inaturalist.org/observations/174474260*

(stredné Slovensko), Belá – východne exponovaný svah [(central Slovakia), Belá (*probably village near Žilina town*), the slope with east exposition], 420 m, 22.VIII.1973, leg. & det. Dvořáková, *SLO*; Rev.: *P. pinetorum*, 12.10.2022, M. Peregrym (*49.2396489ºN, 18.9475208ºE*)

Orava, dolina …(*unreadable)…* osady Široká smerom k obci Kňažia na jz. od Oravského Podzámku [Orava region, … valley Široká village towards Kňažia village, to south-east from Oravský Podzámok village], cca 550 m, 8.VII.1959, leg. & det. J. Futák, *SAV*; Rev.: *P. pinetorum*, 12.10.2022, M. Peregrym (*49.2410567ºN, 19.3296617ºE*)

Široká – Kňažie, v tieni pod smrekmi pospolite [Široká– Kňažie (*probably villages near Oravský Podzámok*), in shadow under the spruces, in groups], 8.8.1959, leg. Zahradníková, *SAV*; Rev.: *P. pinetorum*, 12.10.2022, M. Peregrym (*49.2430233ºN, 19.3317775ºE*)

**29. Spišské vrchy**

Kúpele Ružbachy [Ružbachy spa], 18.6.1882, *collector unreadable*, *BRA*, # 35; Rev.: *P. pinetorum*, 12.10.2022, M. Peregrym (*49.2967664ºN, 20.5653686ºE*)

Prešov Region, Stará Ľubovňa District, near Vyšné Ružbachy village, 49.3021ºN, 20.544ºE, 22.07.2023, Observation by irena_ski: *https://www.inaturalist.org/observations/174295000*

Klein Lomnitz [Klein Lomnitz (*= Lomnička*) village], 1887, leg. V. Vraný, *BRA*, #19, Rev.: *P. pinetorum*, 12.10.2022, M. Peregrym (*49.249448ºN, 20.571173ºE*)

**30a. Šarišská vrchovina**

Prešov Region, Prešov District, near Lipovce village, 49.048206ºN, 20.934451ºE, 01.08.2022, Observation by hemala_vladimir: *https://www.inaturalist.org/observations/130095479*

**30b. Čergov:**

Čergov, Solisko, na lúke pri bývalom salaši, 800 – 900 m, súvislý porast [Čergov Mts, Mt. Solisko, on the meadow near sheep hut, 800 – 900 m, continuous growth], 19-20.IX.1947, leg. & det. J. Májovský, *SLO*; Rev.: *P. pinetorum*, 12.10.2022, M. Peregrym (*49.2126869ºN, 21.1254100ºE*)

Kríže v skupine Čergova, na lúkach a okraji lesov, pieskovec [Mt. Kríže in the Čergov Mts, on the meadows and margin of forests, sandstone], 800 – 900 m, 21.IX.1947, leg. D. Magic, J. Májovský, det. J. Májovský, *SLO*; Rev.: *P. pinetorum*, 12.10.2022, M. Peregrym (*49.2426381ºN, 21.1490817ºE*)

**30c. Nízke Beskydy**

Bardejov [Bardejov town], Stadtweichbildgelände, 1924, 1926, 1927, leg. J. Berganský, det. *unreadable name*, *BRA*, #34, #84, #94, Rev.: *P. pinetorum*, 12.10.2022, M. Peregrym (*49.299103ºN, 21.288476ºE*)

Bardejov [Bardejov town], Ziegeleigelände, 1930, leg. J. Berganský, det. *unreadable name*, *BRA*, #84, Rev.: *P. pinetorum*, 12.10.2022, M. Peregrym (*49.307314ºN, 21.277438ºE*)

Bardejov, Mnichovgelände, 28.V.1929; na pobreží Mníchovského potoka [Bardejov town, on the bank of the Mníchovský potok creek], 18.IV.1929, leg. & det. J. Berganský, *BRA*, #34, Rev.: *P. pinetorum*, 12.10.2022, M. Peregrym (*49.303006ºN, 21.239642ºE*)

Bardejov [Bardejov town], Kameňgelände, 17.VII.1928, leg. J. Berganský, det. *unreadable name*, *BRA*, #34, Rev.: *P. pinetorum*, 12.10.2022, M. Peregrym (*49.285221ºN, 21.242268ºE*)

Bardejov, pri spodnom mlyne [Bardejov town, near the nether mill], 1924, leg. J. Berganský, *BRA*, #34, Rev.: *P. pinetorum*, 12.10.2022, M. Peregrym (*49.295512ºN, 21.284747ºE*)

Bardejov [Bardejov town], Kapitnoka, 24.V.1924, leg. J. Berganský, *BRA*, #34, Rev.: *P. pinetorum*, 12.10.2022, M. Peregrym (*49.281034ºN, 21.268857ºE*)

**31. Bukovské vrchy**

Prešov Region, Snina District, near Kalná Roztoka village, 49.000901ºN, 22.318875ºE, 02.09.2019, Observation by nadler: *https://www.inaturalist.org/observations/156128558*

Prešov Region, Snina District, near Nová Sedlica village, 49.046071ºN, 22.50427ºE, 05.09.2019, Observation by nadler: *https://www.inaturalist.org/observations/161430083*

Prešov Region, Snina District, near Nová Sedlica village, 49.054015ºN, 22.51817ºE, 05.09.2019, Observation by nadler: *https://www.inaturalist.org/observations/160806568*

Prešov Region, Snina District, near Nová Sedlica village, 49.055136ºN, 22.52268ºE, 03.09.2019, Observation by nadler: *https://www.inaturalist.org/observations/159270032*

Prešov Region, Snina District, near Nová Sedlica village, 49.047778ºN, 22.5125ºE, 31.08.2020, Observation by toddpatterson: *https://www.inaturalist.org/observations/58243418*

1. The phytogeographical division of Slovakia is given according to the work of Futák (1984). Localities in individual phytogeographical units are sorted according to the Guidelines for processing the flora of Slovakia (Futák 1973). [↑](#footnote-ref-1)
2. Here and further: if there are no geographic coordinates in the original source, then they have been identified and indicated (in italics) approximately with the using of Google map (*https://www.google.com/maps*) for the purpose of mapping of known locations of *Pteridium* species. If hills are indicated as localities, then the coordinate is the top of that hill, if a village is indicated, then the coordinate is the center of the village, if in all these cases something specific is not noted. [↑](#footnote-ref-2)
3. The text in italics is our notes or guesses for a more detailed explanation of the location. [↑](#footnote-ref-3)
