# Supplementary material for: A new insight on the genus Pteridium (Dennstaedtiaceae) in Europe based on a revision in the flora of Slovakia
Source: Bot Stud. 2024 Aug 14;65:24. doi: 10.1186/s40529-024-00423-1 (PMC11324619; doi:10.1186/s40529-024-00423-1)
Supplement: Supplementary file 2 — Supplementary Material 2. Confirmed locations of Pteridium aquilinum subsp. pinetorum (C.N. Page & R.R. Mill) J.A. Thomson in Austria, Czechia, Hungary and Romania [file 40529_2024_423_MOESM2_ESM.docx]

Supplementary materials B

**Confirmed locations of *Pteridium aquilinum* subsp. *pinetorum* (C.N. Page & R.R. Mill) J.A. Thomson in Austria, Czechia, Hungary and Romania**

**Austria^[[1]](#footnote-1)^**:

1. *Eupteris aquilina* (L.) New., Austria inferior. In silvis “Durchlass” inter Lunz et Lackenhof. Alt. ca 650-700 m. s. m., 12 jul. 1923, Dr. Boros, *BP*, #29401; Rev.: *Pteridium pinetorum* C.N. Page & R.R. Mill, 6.10.2022, M. Peregrym;

2. *Pteris aqulina* L., Wien, 19 Aug. 1860, Bayer, *BP*, #29474; Rev.: *Pteridium pinetorum* C.N. Page & R.R. Mill, 6.10.2022, M. Peregrym

3. *Pteridium aquilinum* (L.) Kuhn. var. *glabrum* (Hook.), Comit. Vas. Borostyánkő, 4.VI.1911, Dr. Filarszky, Dr. Kümmerle et Dr. Zahlbruckner, *BP*, #29499; Rev.: *Pteridium pinetorum* C.N. Page & R.R. Mill, 6.10.2022, M. Peregrym;

4. *Pteridium aquilinum* (L.) Kuhn. Lunz. 1905. 20.IX. Leg.: Dr. Kümmerle, *BP*, #29553; Rev.: *Pteridium pinetorum* C.N. Page & R.R. Mill, 6.10.2022, M. Peregrym;

5. *Pteris aquilina* L., Habitat in silvis lucidis ad Pinkofő et Sinnersdorf, 1904 jul. 23. Leg. Dr. Simonkai Lajos, *BP*, #29617; Rev.: *Pteridium pinetorum* C.N. Page & R.R. Mill, 6.10.2022, M. Peregrym;

6. *Pteris aquilina* L., Troppberg bei Gablitz, 29 aug. 1861, J.B. Holzinger, *SLO*; Rev.: *Pteridium pinetorum* C.N. Page & R.R. Mill, 12.10.2022, M. Peregrym;

7. *Pteris aquilina* L., in mont. apric. Vien: (*?*)ekawinkel, asi 15 aug.1860, J.N. Bayer, *SLO*; Rev.: *Pteridium pinetorum* C.N. Page & R.R. Mill, 12.10.2022, M. Peregrym.

**Czechia**:

1. *Pteridium aquilinum* Kuhn, Moravia centralis: in silvaticis prope Šlapanice, 13.IX.1925, leg. J. Bílý, *BP*, #29467; Rev.: *Pteridium pinetorum* C.N. Page & R.R. Mill, 6.10.2022, M. Peregrym;

2. *Pteridium aquilinum* (L.) Kuhn, Mostek u Hostinného, na okraji lesa, 550 m, 20.8.1947, leg. & det. V. Kavka, *BRA*, #48;

3. *Pteris aquilina*, Bud. 1916, českobudějovické polesí, leg. A. Hejsek, *BRA*, #50;

4. *Pteridium aquilinum* (L.) Kuhn, Sušicko: Šimanov – okraj (*unreadable*) jižnĕ osady. Die: 11.8.1967, Leg. J. Vaněček, *BRA*;

5. *Pteridium aquilinum* Kuhn, Olomouc: Jungwald b. Weska, VIII.1930, leg. Lang, *SLO*; Rev.: *Pteridium pinetorum* C.N. Page & R.R. Mill, 12.10.2022, M. Peregrym.

**Hungary:**

1. *Pteridium aquilinum* (L.) Kuhn, Bakony, (*unreadable*) Király mellett, Dr. Pillitz Bénő, *BP*, #29291; Rev.: *Pteridium pinetorum* C.N. Page & R.R. Mill, 6.10.2022, M. Peregrym;

2. *Pteridium aquilinum* (L.) Kuhn., cott. Szabolcs, in silva arenosa Korhányi-erdő pr. pag. Nyirbakta, 26.VI.1932, Leg. Dr. R. de Soó, *BP*, #29307 / In arenosis dumetosis, 4 jun. 1927. Alt. s. m. met. ca: 140, *BP*, #29408; Rev.: *Pteridium pinetorum* C.N. Page & R.R. Mill, 6.10.2022, M. Peregrym;

3. *Pteridium aquilinum* (L.) Kuhn., In Pinetum silvestre cult. procul viam inter pagum Ófehértó et Baktalórántházá comit. Szabolcs, 16.VI.1949, Leg. Ujhelyi J., Baksay L., *BP*, #29308, #29310;

4. *Eupteris aquilina* (L.) Newman f. *vulgaris* (Borb.), Comit. Pest. In silvis caeduis in latere boreali montis Nagy Kopaszhegy ad pagum Piliscsaba. Altit. ca 300 m., sol. calc., Septembr. 1916, leg. F. Filarszky et J.B. Kűmmerle, *BP*, #29312, #29328, #29634, #49185; Rev.: *Pteridium pinetorum* C.N. Page & R.R. Mill, 6.10.2022, M. Peregrym;

5. *Pteridium aquilinum* (L.) Kuhn., cott. Szabolcs, in silva arenosa “Külső Guth-erdő”, 5.VI.1931, *BP*, #29315;

6.*Pteridium aquilinum* (L.) Kuhn., Nyírvasvári: Bátori-erdő. Szatmár megye, 1946, VI.10., *BP*, #29317; Rev.: *Pteridium pinetorum* C.N. Page & R.R. Mill, 6.10.2022, M. Peregrym;

7. *Pteridium aquilinum* (L.) Kuhn, Gömör m., Putnok, (*unreadable*), 300 m s.m., 1924.VII.8, *BP*, #29316;

8. *Pteridium aquilinum* (L.) Kuhn., Bélatanya (= Ócskatanya) Bátorligetnél. Szabolcs megye, 1946, VI.10., *BP*, #29318; Rev.: *Pteridium pinetorum* C.N. Page & R.R. Mill, 6.10.2022, M. Peregrym;

9. *Pteridium aquilinum* (L.) Kuhn., Tiborszállás: Vadaskert. (Szatmár m.), 1946, VI.26., *BP*, #29321; Rev.: *Pteridium pinetorum* C.N. Page & R.R. Mill, 6.10.2022, M. Peregrym;

10. *Pteridium aquilinum* (L.) Kuhn., In montibus Bakonyhegység, in silva Zabolaerdő. Comit. Veszprém. 7.V.1950. (*unreadable*), *BP*, #29322; Rev.: *Pteridium pinetorum* C.N. Page & R.R. Mill, 6.10.2022, M. Peregrym;

11. *Pteridium aquilinum* (L.) Kuhn., cott. Szatmár, in silva arenosa Kőrmei-erdő (Fényi) pr. pag. Zezőfeny, 1.VII.1948, S.J.S., *BP*, #29323, #29372; Rev.: *Pteridium pinetorum* C.N. Page & R.R. Mill, 6.10.2022, M. Peregrym;

12.*Pteridium aquilinum* (L.) Kuhn., In montibus Sátorhegység, in monte Paphegy, supra vicum Sompatakinagyhuta. comit. Zemplén, 14.VII.1950, Leg. (*unreadable*), *BP*, #29324; Rev.: *Pteridium pinetorum* C.N. Page & R.R. Mill, 6.10.2022, M. Peregrym;

13. *Pteridium aquilinum* (L.) Kuhn., Fábiánházá: Mérki-erdő (Szatmár m.), 1946, VI.26., Papp József, *BP*, #29325; Rev.: *Pteridium pinetorum* C.N. Page & R.R. Mill, 6.10.2022, M. Peregrym;

14. *Pteridium aquilinum* (L.) Kuhn., In montibus Sátorhegység, in querceto montis Gillevárierdő, comit. Zemplén, 14.VII.1950, Leg. (*unreadable*), *BP*, #29326; Rev.: *Pteridium pinetorum* C.N. Page & R.R. Mill, 6.10.2022, M. Peregrym;

15. *Pteridium aquilinum* (L.) Kuhn., Fábiánházá: Cservágás (Mérki-erdő), Nyírség, Szatmár megye, 1946, VI.26. Papp József, *BP*, #29327; Rev.: *Pteridium pinetorum* C.N. Page & R.R. Mill, 6.10.2022, M. Peregrym;

16. *Pteridium aquilinum* (L.) Kuhn, Comit. Abauj-Torna. Mt. Sátorhegység. In fagetis saxosis sub radicibus montis Nagy-Péter-Ménkő supra vallem Őrdőgvőlgy prope pagum Pálháza, alt. cca 500 m.s.m., 6.IX.1951, *BP*, #29338; Rev.: *Pteridium pinetorum* C.N. Page & R.R. Mill, 6.10.2022, M. Peregrym;

17. *Pteris aquilina* L., in silvis lucidis ad Piliscsaba, 1904. okt. 2., Dr. Simonkai Lajos, *BP*, #29354; Rev.: *Pteridium pinetorum* C.N. Page & R.R. Mill, 6.10.2022, M. Peregrym;

18. *Pteridium aquilinum* (L.) Kuhn., Abauj-Torna m., Fűzérkomlós, erdőszélén. 1933.VI.24. S. Jávorka, *BP*, #29355; Rev.: *Pteridium pinetorum* C.N. Page & R.R. Mill, 6.10.2022, M. Peregrym;

19. *Pteridium aquilinum* (L.) Kuhn, Comit. Szabolcs: (*unreadable*) ad prope Nyirbátor, VIII/20.912, Leg. Dr. Kümmerle et Timkő, *BP*, #29356; Rev.: *Pteridium pinetorum* C.N. Page & R.R. Mill, 6.10.2022, M. Peregrym;

20. *Pteris aquilina* L., In silvis ad Pilis-Szántó, 1904. X. 2., Leg. (*unreadable*), *BP*, #29359; Rev.: *Pteridium pinetorum* C.N. Page & R.R. Mill, 6.10.2022, M. Peregrym;

21. *Pteridium aquilinum* (L.) Kuhn, Comit. Vesprém, Montis Bakony, in valle Cuha, 23.V.1928., *BP*, #29360, #29452, #29453; Rev.: *Pteridium pinetorum* C.N. Page & R.R. Mill, 6.10.2022, M. Peregrym;

22. *Pteridium aquilinum* (L.) Kuhn, Montes Bakony: in m. Tobán ad pagum Eplény, VI.1933, *BP*, #29364; Rev.: *Pteridium pinetorum* C.N. Page & R.R. Mill, 6.10.2022, M. Peregrym;

23. *Pteridium aquilinum* (L.) Kuhn, Comit. Zala: in m. Halápi hegy, 27.IV.1913, *BP*, #29365; Rev.: *Pteridium pinetorum* C.N. Page & R.R. Mill, 6.10.2022, M. Peregrym;

24. *Pteridium aquilinum* (L.) Kuhn, Montes Bakony: in valle Hódosér, 6.VI.1932, *BP*, #29369, #29370; Rev.: *Pteridium pinetorum* C.N. Page & R.R. Mill, 6.10.2022, M. Peregrym;

25. *Pteris aquilina*, Pilisszentiván m., (*unreadable*), 1928.VIII.2., leg. Rosembersky, *BP*, #29371; Rev.: *Pteridium pinetorum* C.N. Page & R.R. Mill, 6.10.2022, M. Peregrym;

26. *Pteridium aquilinum* (L.) Kuhn., cott. Szabolcs, in silvaticis arenosis ad viam ferream pr. praed. Külsőguth, 27.V.1937, Dr. M. Ujvárosi, *BP*, #29373; Rev.: *Pteridium pinetorum* C.N. Page & R.R. Mill, 6.10.2022, M. Peregrym;

27. *Pteridium aquilinum* (L.) Kuhn., Comit. Szabolcs. Territ. Nyírbátor. In querceto prope Kispiricse. Alt. cca. 140 m, 1.IX.1932, *BP*, #29375; Rev.: *Pteridium pinetorum* C.N. Page & R.R. Mill, 6.10.2022, M. Peregrym;

28. *Eupteris aquilina* (L.) New., Comit. Szabolcs. In dumetosis ad Bátorliget prope Nyirbátor. Alt. s. m. met. ca: 140, 31 aug. 1920. *BP*, #29379, #29415; Rev.: *Pteridium pinetorum* C.N. Page & R.R. Mill, 6.10.2022, M. Peregrym;

29. *Pteridium aquilinum* (L.) Kuhn., Bakony. Fenyőfő mellet, Comit. Veszprém. 1952.VII.22., *BP*, #29383; Rev.: *Pteridium pinetorum* C.N. Page & R.R. Mill, 6.10.2022, M. Peregrym;

30. *Pteridium aquilinum* (L.) Kuhn., Sátorhegyek. Vajdavőlgy felett, a Vádasztető alján. (*unreadable*). 1952. aug. 27., *BP*, #29384; Rev.: *Pteridium pinetorum* C.N. Page & R.R. Mill, 6.10.2022, M. Peregrym;

31. *Pteridium aquilinum*, „Székierdő” prope Bakonygyepes (Comit. Veszprém), 14.VI.1977, Leg.: (*unreadable*), Det.: (*unreadable*), *BP*, #29393; Rev.: *Pteridium pinetorum* C.N. Page & R.R. Mill, 6.10.2022, M. Peregrym;

32. *Pteris aquilina* L., Comit. Pest. In silvis inter mont. „Dobogókő” et „Kétbükkfanyeger.” Alt. s. m. met. ca: 650, 18 sept. 1918, *BP*, #29399, #29400, #29510;

33. *Eupteris aquilina* (L.) New. Comit. Vas. In declivibus montis prope “Hétforrás” ad urben Kőszeg. Alt. ca 400-met. s.m., 10 jul. 1915, Legit: Simonyi Semadam Sándor jun., *BP*, #29405; Rev.: *Pteridium pinetorum* C.N. Page & R.R. Mill, 6.10.2022, M. Peregrym;

34. *Pteridium aquilinum* (L.) Kuhn., Comit. Szabolcs. In collibus arenosis ad Mailáthtelep inter pag. Ófehértó et Nyírbakta, Alt. s. m. met. ca: 150, 18 sept. 1926. *BP*, #29409;

35. *Pteridium aquilinum* (L.) Kuhn., Comit. Szabolcs. In agris arenosis (*unreadable*) ad Forgáts-tanyák prope Tornyospálca. Alt. s. m. met. ca: 100, 8 sept. 1925. *BP*, #29412; Rev.: *Pteridium pinetorum* C.N. Page & R.R. Mill, 6.10.2022, M. Peregrym;

36. *Pteridium aquilinum* (L.) Kuhn., Comit. Szabolcs. In betulete “Pálca-erdő” prope Tornyospálca. Alt. s. m. met. ca: 100, 8 sept. 1925. *BP*, #29413; Rev.: *Pteridium pinetorum* C.N. Page & R.R. Mill, 6.10.2022, M. Peregrym;

37. *Eupteris aquilina* (L.) New., Comit. Szabolcs. In dumetis “Kispiricsoi erdő” prope Nyirbátor. Alt. s. m. met. ca: 150, 29 jun. 1922. *BP*, #29414; Rev.: *Pteridium pinetorum* C.N. Page & R.R. Mill, 6.10.2022, M. Peregrym;

38. *Eupteris aquilina* (L.) Newman f. *vulgaris* (Borb.) Kümm, Comit. Vas. In dumetosis jugi inter montes Budi Riogel et Szarvaskő, prope pagum Rohono, Alt. s. m. met. ca: 6-800, 26 sept. 1920, *BP*, #29418; Rev.: *Pteridium pinetorum* C.N. Page & R.R. Mill, 6.10.2022, M. Peregrym;

39. *Pteridium aquilinum* (L.) Kuhn. f. *glabrum* (Hook.) Comit. Hajdu. In silva “Savoskut erdő” inter Hajdusámson et Hajduhadház, Alt. s. m. met. ca: 150, 7 jun. 1924, *BP*, #29419; Rev.: *Pteridium pinetorum* C.N. Page & R.R. Mill, 6.10.2022, M. Peregrym;

40. *Pteridium aquilinum* (L.) Kuhn. f. *glabrum* Hook., Comit. Pest. Ad fontem Háromforrások prope pag. Pilisszentkereszt, 12.VI.1916, leg. Ő. Rosemberszky, *BP*, #29433; Rev.: *Pteridium pinetorum* C.N. Page & R.R. Mill, 6.10.2022, M. Peregrym;

41. *Pteris aquilina* L. A Mamut alján. 1882. Cserni B., *BP*, #29495; Rev.: *Pteridium pinetorum* C.N. Page & R.R. Mill, 6.10.2022, M. Peregrym;

42. *Pteris aquilina* L. *glabra*, Kőszeg mellett. Vas megye. Leg. D. Waisbecker A., *BP*, #29501; Rev.: *Pteridium pinetorum* C.N. Page & R.R. Mill, 6.10.2022, M. Peregrym;

43. *Pteridium aquilinum* (L.) Kuhn. Comit. Baranya: in (*unreadable*) montis Jakabhegy ad pag. Pellérd, 7.VI.1922, Kümmerle et Jávorka, *BP*, #29503; Rev.: *Pteridium pinetorum* C.N. Page & R.R. Mill, 6.10.2022, M. Peregrym;

44. *Pteridium aquilinum* (L.) Kuhn var. *glabrum* (Hook.), Comit. Zala: in arenosis ad pedem montis Haláp ad pagum Haláp, alt. 200 m., IV.27.1913, leg. (*unreadable*), *BP*, #29505, #29506; Rev.: *Pteridium pinetorum* C.N. Page & R.R. Mill, 6.10.2022, M. Peregrym;

45. *Pteridium aquilinum* (L.) Kuhn, Dédes (Borsod m.), 1906 sept. 19., (*unreadable*), *BP*, #29518; Rev.: *Pteridium pinetorum* C.N. Page & R.R. Mill, 6.10.2022, M. Peregrym;

46. *Pteridium aquilinum* (L.) Kuhn, Comit. Zala, Pr. pg. Sárchida. *Querceto-Carpinetum* in silv. „Baki erdő”. 15 mai. 1953, *BP*, #29552; Rev.: *Pteridium pinetorum* C.N. Page & R.R. Mill, 6.10.2022, M. Peregrym;

47. *Pteris aquilina* L., Rétaguhegy alján, 1877. Sept., (*unreadable*), *BP*, #29610; Rev.: *Pteridium pinetorum* C.N. Page & R.R. Mill, 6.10.2022, M. Peregrym;

48. *Pteris aquilina* L., Kőszeg, 1883, (*unreadable*), *BP*, #29623; Rev.: *Pteridium pinetorum* C.N. Page & R.R. Mill, 6.10.2022, M. Peregrym;

49. *Pteridium aquilinum* (L.) Kuhn, Comit. Samogy: in pineto „Pati erdő” ad pag. Kaposmérő, 22.IX.1932, S. Jávorka, *BP*, #29624; Rev.: *Pteridium pinetorum* C.N. Page & R.R. Mill, 6.10.2022, M. Peregrym;

50. *Pteridium aquilinum* (L.) Kuhn, Bakony: (*unreadable*) supra p. Porva, 1927.VI.5, S. Jávorka, *BP*, #29627; Rev.: *Pteridium pinetorum* C.N. Page & R.R. Mill, 6.10.2022, M. Peregrym;

51. *Pteridium aquilinum* (L.) Kuhn, Comit. Veszprém. Montes Bakony: ad pagum Fenyőfő, sol. calc., IV.28.1927, leg. & det. (*unreadable*), *BP*, #29628; Rev.: *Pteridium pinetorum* C.N. Page & R.R. Mill, 6.10.2022, M. Peregrym;

52. *Pteridium aquilinum* (L.) Kuhn f. *umbrosum*, Montes Bakony: in (*unreadable*) m. Kőröshegy supra (*unreadable*) – 5.VII.1924, S. Jávorka, *BP*, #29629; Rev.: *Pteridium pinetorum* C.N. Page & R.R. Mill, 6.10.2022, M. Peregrym;

53. *Pteris aquilina* L., In silvis as pagum „Pilis Csaba”. Com. Pest, 26.VI.1908, leg. Szombathy Kálmán, *BP*, #29638, #29639; Rev.: *Pteridium pinetorum* C.N. Page & R.R. Mill, 6.10.2022, M. Peregrym;

54. *Pteridium aquilinum* (L.) Kuhn, Comitat Zala, In fagetis pr. pag. Lovászi, Alt. cca. 200 m, 30.8.1954, Determinavit: Á. Károlyi, *BP*, #29777; Rev.: *Pteridium pinetorum* C.N. Page & R.R. Mill, 6.10.2022, M. Peregrym;

55. *Pteris aquilina* Sw., Mohos tó, a (*unreadable*), 1892, szept. 3., *BP*, #29801; Rev.: *Pteridium pinetorum* C.N. Page & R.R. Mill, 6.10.2022, M. Peregrym;

56. *Pteridium aquilinum* Kh, Tót Hegymeg. Aug. 1885, leg. Aladár Richter, *BP*, #29819; Rev.: *Pteridium pinetorum* C.N. Page & R.R. Mill, 6.10.2022, M. Peregrym;

57. *Eupteris aquilina* (L.) New., Comit. Zala/ Ad rivum “Viszlói patak” prope Tapolca. Alt. s. m. met. ca: 120. 26. aug. 1923, *BP*, #29?03; Rev.: *Pteridium pinetorum* C.N. Page & R.R. Mill, 6.10.2022, M. Peregrym;

58. *Pteridium aquilinum* (L.) Kuhn., Com. Somogy, prope pagum Darány, “Nagyberek”, 1978, Jul., Leg. L. Hably, F. Németh, T. Szerdahelyi, Det. M. Kertész *BP*, #45805, #45845; Rev.: *Pteridium pinetorum* C.N. Page & R.R. Mill, 6.10.2022, M. Peregrym;

59. *Pteridium aquilinum* (L.) Kuhn., Dobogókő, in pratis silvaticis, alt. ca 680 m s.m., 1937.20.V., (*unreadable*), *BP*, #49076; Rev.: *Pteridium pinetorum* C.N. Page & R.R. Mill, 6.10.2022, M. Peregrym;

60. *Pteris aquilina* L., P. Szántő és P. Czaba (*unreadable*), 1890 jul. 6., (*unreadable*), *BP*, #49107; Rev.: *Pteridium pinetorum* C.N. Page & R.R. Mill, 6.10.2022, M. Peregrym;

61. *Pteridium aquilinum* (L.) Kuhn, In pratis sub monte Kőrishegy prope Zircz, Legit: 1920 V/25 Dr. Tuzson, Determ: 1920 VI/9 Andreánszky, *BP*, #49184; Rev.: *Pteridium pinetorum* C.N. Page & R.R. Mill, 6.10.2022, M. Peregrym;

62. *Pteridium aquilinum* (L.) Kuhn, Pest m., Pilisvőrősvár: Pilisi-híd hegycsoport, “Északi-vonulat”, Az északkeleti tetőn, leégett „kopárfásítás” helyén, Vágásnővényzet. Alt. 220 m, 2001.VII.19, Leg. & Det. Bőhm É.I., *BP*, #51166; Rev.: *Pteridium pinetorum* C.N. Page & R.R. Mill, 6.10.2022, M. Peregrym;

63. *Pteridium aquilinum* (L.) Kuhn., Comit. Szabolcs., “Júlia-liget”, in margine Betuleti “Zsibolya” nominate pr. pag. Piricse. 3/VI.1998. Dr. L. Felföldy, *BP*, #51367; Rev.: *Pteridium pinetorum* C.N. Page & R.R. Mill, 6.10.2022, M. Peregrym;

64. *Pteridium aquilinum* (L.) Kuhn., Comit. Abaúj, in prato montano „Bohó-rét” pr. pag. Regéc. 24/VI.1979., Dr. Á. Hegedűs, *BP*, #51460; Rev.: *Pteridium pinetorum* C.N. Page & R.R. Mill, 6.10.2022, M. Peregrym;

65. *Pteridium aquilinum* (L.) Kuhn, Komárom-Esztergom m., Várgesztes: Vadász-dombok Ny-I része, laposon; tőlgyesben, 2002.V.19, Barina Z. – Pifkó D., *BP*, #51702; Rev.: *Pteridium pinetorum* C.N. Page & R.R. Mill, 6.10.2022, M. Peregrym;

66. *Pteridium aquilinum* (L.) Kuhn, Comit. Veszprém, in betuletis ad stationem ferroviae Uzsabánya. 11/VI.1965. F. Radics, *BP*, #51722; Rev.: *Pteridium pinetorum* C.N. Page & R.R. Mill, 6.10.2022, M. Peregrym;

67. *Pteridium aquilinum* (L.) Kuhn, Pest megye, Pilis hegység, Pilisszántó: Hosszú-hegy, a 485 m-es csúcsnál, savanyú talajon. 2006. június 11. Somlyay Lajos, *BP*, #52291; Rev.: *Pteridium pinetorum* C.N. Page & R.R. Mill, 6.10.2022, M. Peregrym;

68. *Pteridium aquilinum* (L.) Kuhn, Locus natalis: In silva solo arenario, prope pagum Aporliget, com. Szabolcs-Szatmár. In associatione: *Festuco-Quercetum roboris.* 1.VII.1957. Leg.: Jeney, *BP*, #52473; Rev.: *Pteridium pinetorum* C.N. Page & R.R. Mill, 6.10.2022, M. Peregrym;

69. *Pteridium aquilinum* (L.) Kuhn, Locus natalis: In silva umbrosa solo arenosohumidoso, pr. m. Debrecen (*unreadable*) alt. cca 8 m s.m. In associatione: *Querceto-Convallarietum*. 16.VI.1954 Legit: Jeney, *BP*, #52474; Rev.: *Pteridium pinetorum* C.N. Page & R.R. Mill, 6.10.2022, M. Peregrym;

70. *Pteridium aquilinum* (L.) Kuhn, Locus natalis: In silva pineta culta solo arenoso sicco, prope pagum Aporliget, Fenyí-erdö, comit. Szabolcs-Szatmár. 1955 VI.27. Legit: Jeney, *BP*, #52475; Rev.: *Pteridium pinetorum* C.N. Page & R.R. Mill, 6.10.2022, M. Peregrym;

71. *Pteridium aquilinum* L., Locus natalis: In silva querceta arenosa sicca, prope pagum Guthi-erdő, comit. Hajdu-Bihar. In associatione: *Festuco-Quercetum pteridetosum* (*unreadable*). 1956. jul. 7., *BP*, #52476, #52477; Rev.: *Pteridium pinetorum* C.N. Page & R.R. Mill, 6.10.2022, M. Peregrym;

72. *Pteridium aquilinum* L., Pest megye, Pilisvőrősvár: “Zajnát-hegyek”, leégett feketefenyő ültetvény helyén, vágásnővényzetben, tőmegesen. 2001. július 19. Bőhm É.I., *BP*, #52649; Rev.: *Pteridium pinetorum* C.N. Page & R.R. Mill, 6.10.2022, M. Peregrym.

**Romania:**

1. *Pteridium aquilinum* (L.) Kuhn., Kolozsvár, in silvaticis decl. ad EKE-menedékházak mt. Bűkk, Alt. s. m. met. ca: 600, 17.VI.1942, Dr. M. Ujvárosi, *BP*, #29320; Rev.: *Pteridium pinetorum* C.N. Page & R.R. Mill, 6.10.2022, M. Peregrym;

2.*Pteridium aquilinum* (L.) Kuhn., Transsilvania: in silvis ad Nagyenyed, 13.VII.1904, leg. R. Repaics, *BP*, #29367; Rev.: *Pteridium pinetorum* C.N. Page & R.R. Mill, 6.10.2022, M. Peregrym;

3. *Pteridium aquilinum* (L.) Kuhn, Comit. Máramaros. In rupestribus (*unreadable*) vőlgy supra pag. Szaplonca, alt. cca 900 m. s. m., 24.VII.1941, *BP*, #29376; Rev.: *Pteridium pinetorum* C.N. Page & R.R. Mill, 6.10.2022, M. Peregrym;

4. *Pteridium aquilinum* (L.) Kuhn., in silva Várerdő prope Gyalu, com.: Kolozs. Alt. s. m. met. ca: 500, 18 jul. 1942, Dr. J. Igmándy, *BP*, #29391, Rev.: *Pteridium pinetorum* C.N. Page & R.R. Mill, 6.10.2022, M. Peregrym;

5. *Pteridium aquilinum* (L.) Kuhn, Rétyi Nyir. 1942.8.30, *BP*, #29395, #29396, Rev.: *Pteridium pinetorum* C.N. Page & R.R. Mill, 6.10.2022, M. Peregrym;

6. *Pteridium aquilinum* (L.) Kuhn, Rétyi Nyír. (*unreadable*). 1907. jul. 27. (*unreadable*), *BP*, #29489; Rev.: *Pteridium pinetorum* C.N. Page & R.R. Mill, 6.10.2022, M. Peregrym;

7. *Pteridium aquilinum* (L.) Kuhn, Comit. Hunyad: in silvaticis adp. Kimpulujnyág sub alp. Retyczát. 909. VII/10. Dr. Jávorka, *BP*, #29490; Rev.: *Pteridium pinetorum* C.N. Page & R.R. Mill, 6.10.2022, M. Peregrym;

8. *Pteridium aquilinum* (L.) Kuhn, Comit. Csík, Tusnad. Substrat: Trachyt. Hőhe: 900 m. 16.8.1896. legit: (*unreadable*), *BP*, #29492; Rev.: *Pteridium pinetorum* C.N. Page & R.R. Mill, 6.10.2022, M. Peregrym;

9. *Pteridium aquilinum* (L.) Kuhn, Comit. Csík, in (*unreadable*) monte öcsém prope pag. Balínbánya. VII.25.1915, leg. Kümmerle et Jávorka, *BP*, #29493; Rev.: *Pteridium pinetorum* C.N. Page & R.R. Mill, 6.10.2022, M. Peregr1ym;

10. *Pteridium aquilinum* Kuhn, Csík-Gyemesen Csíkvarmegyeben. 1902. jul. 5, Kümmerle (*unreadable*), *BP*, #29494; Rev.: *Pteridium pinetorum* C.N. Page & R.R. Mill, 6.10.2022, M. Peregrym;

11. *Pteridium aquilinum* (L.) Kuhn. In catena montis “Hargitta” supra Balnea-Homoród, in monte “Czekendtető” dioto Com. Udvarhely. Legit 14 Jun. 1907, L. de Thaisz., *BP*, #29651, Rev.: *Pteridium pinetorum* C.N. Page & R.R. Mill, 6.10.2022, M. Peregrym;

12. *Pteridium aquilinum* (L.) Kuhn, Transsilvania, distr. Turda. In caeduis prope pag. Filea de jos, alt. cca 450 m s.m. – 5 Sept. 1938. leg. E. Ghişa; *BP*, #29655, Rev.: *Pteridium pinetorum* C.N. Page & R.R. Mill, 6.10.2022, M. Peregrym;

13. *Pteridium aquilinum* (L.) Kuhn. monstr. *inaequale* Hrieg., Comit. Háromszék. In silvis inter (*unreadable*) Tusnád et lacum Szt.-Anna tó; alt. 900 m., sol. trachyt. 27.07.1915, Leg. Dr. Kümmerle et Dr. Jávorka, *BP*, #29824; Rev.: *Pteridium pinetorum* C.N. Page & R.R. Mill, 6.10.2022, M. Peregrym;

14. *Pteris aquilina* L., in monte (*unreadable*) prope pag. Dubova ad Dunubium inferiorem. 1873. aug. 19, V. Bobás, *BP*, #49106; Rev.: *Pteridium pinetorum* C.N. Page & R.R. Mill, 6.10.2022, M. Peregrym;

15.*Pteridium aquilinum* (L.) Kuhn., Vöröstoronyi szoros. 1892. VII., leg. (*unreadable*), *BP*, #29809, Rev.: *Pteridium pinetorum* C.N. Page & R.R. Mill, 6.10.2022, M. Peregrym.

1. The country order is alphabetical. The order of herbarium specimens is in the alphabetical order of herbarium acronyms, and then, to their inventory numbers. [↑](#footnote-ref-1)
